# Integrated acupuncture-pharmacotherapy for perimenopausal insomnia: a systematic review and meta-analysis

**DOI:** 10.3389/fneur.2025.1633794

**Published:** 2025-08-20

**Authors:** Boxiang Yang, Shengwen Jiang, Yubo Teng, Yiming Wang, Jingyi Zhang, Chang Gao, Chunhua Song

**Affiliations:** ^1^School of Graduate, Heilongjiang University of Chinese Medicine, Harbin, China; ^2^School of Basic Medical Science, Shenyang Medical College, Shenyang, China; ^3^Department of Acupuncture X, Heilongjiang Academy of Traditional Chinese Medicine, Harbin, China; ^4^Department of Acupuncture IX, The Second Affiliated Hospital of Heilongjiang University of Chinese Medicine, Harbin, China

**Keywords:** acupuncture, pharmacotherapy, perimenopause, insomnia, systematic review, meta-analysis

## Abstract

**Objective:**

Insomnia is a prevalent symptom among perimenopausal women, mainly attributed to estrogen-progesterone imbalance and neuropsychiatric factors, significantly impacting their quality of life. This article seeks to systematically evaluate the efficacy of integrated acupuncture-pharmacotherapy (AP) in treating perimenopausal insomnia (PMI), offering new insights for the management of insomnia in women.

**Methods:**

Searches were conducted in 8 databases: PubMed, Web of Science (WOS), Cochrane Library, Embase, China National Knowledge Infrastructure (CNKI), China Biology Medicine Disc (CBM), Wanfang Academic Journal Full-text Database (Wanfang), and Chongqing VIP Database (CQVIP). Database searches extended through August 1, 2024. Endnote 20 was used to build the database and screen for eligible randomized controlled trials (RCTs). The efficacy of AP for PMI were demonstrated by assessing 3 primary outcome measures (Effective rate, Hamilton Anxiety Scale [HAMA], Traditional Chinese Medicine Syndromes [TCMS]) and 5 secondary outcome measures (Pittsburgh Sleep Quality Index [PSQI], Modified Kupperman Index [KMI], Luteinizing Hormone [LH], Follicle-Stimulating Hormone [FSH], Estradiol [E_2_]). The risk of bias was assessed according to the *Cochrane Handbook for Systematic Reviews of Interventions*. Data analysis was performed using RevMan 5.4 and StataMP 15.0. Subgroup or sensitivity analysis was applied as necessary to address issues of heterogeneity. Regression analysis was used to determine whether the division of potential subgroups is reasonable. The evidence quality level was evaluated using the GRADEprofiler following the GRADE approach.

**Results:**

A total of 12 eligible studies comprising 969 PMI cases were ultimately included in this meta-analysis. Pooled results indicated AP had statistically significant benefits for PMI: Efficacy (Effective rate [RR = 1.22, 95% CI (1.13, 1.30), *Z* = 3.88, *p* < 0.00001]), Scores (HAMA [MD = −3.26, 95% CI (−3.79, −2.73), *Z* = 12.06, *p* < 0.00001]), TCMS [MD = −0.98, 95% CI (−1.21, −0.74), *Z* = 7.99, *p* < 0.00001], PSQI [MD = −3.12, 95% CI (−4.21, −2.03), *Z* = 5.63, *p* < 0.00001], KMI [MD = −3.96, 95% CI (−5.78, −2.15), *Z* = 4.28, *p* < 0.0001], and Hormone levels LH [MD = −10.16, 95% CI (−16.41, −3.91), *Z* = 3.18, *p* = 0.001 < 0.05], FSH [MD = −8.65, 95% CI (−13.67, −3.64), *Z* = 3.39, *p* = 0.0007 < 0.05], E_2_ [MD = 15.87, 95% CI (10.16, 21.58), *Z* = 5.45, *p* < 0.00001].

**Conclusion:**

AP demonstrates significant efficacy in treating PMI patients, offering an innovative integrative therapy with substantial clinical value. Future studies should involve more large-scale, multicenter RCTs with long-term follow-up.

**Systematic review registration:**

https://www.crd.york.ac.uk/PROSPERO/view/CRD42024579691.

## Introduction

Perimenopause represents a critical transitional period in a woman’s life, marking the shift from reproductive capability to menopause. According to the STRAW +10 criteria, it is characterized by a gradual decline in ovarian function and is divided into 2 substages: early perimenopause (Stage −2), marked by increased menstrual cycle variability and elevated early follicular phase FSH, and late perimenopause (Stage −1), defined by amenorrhea lasting≥60 days, significantly elevated FSH levels, and markedly reduced anti-Müllerian hormone (AMH) and antral follicle count (AFC) (1). This stage is accompanied by pronounced hormonal fluctuations and multisystem dysregulation, collectively manifesting as perimenopausal syndrome (PMS) (2). Among the most common and distressing symptoms is insomnia, affecting 30–50% of perimenopausal women. Emerging evidence suggests that the impact of insomnia extends beyond quality of life impairment, exerting a significant influence on cardiovascular and cognitive health through multifactorial pathways. Chronic sleep disturbances may contribute to dysregulation of the hypothalamic–pituitary–adrenal (HPA) axis, heightened systemic inflammation, and increased sympathetic nervous system activity, all of which can promote atherogenesis and elevate the risk of myocardial infarction (MI). A longitudinal population-based study using data from the UK Household Longitudinal Study found that individuals with elevated levels of social dysfunction and anhedonia—core dimensions of psychological distress—had a significantly increased risk of developing MI over a 10-year period (3). Moreover, in patients already diagnosed with coronary heart disease (CHD), significantly worse scores across multiple domains of mental health, including depression, anxiety, and loss of confidence, were observed (4). These findings suggest a bidirectional interplay between cardiovascular vulnerability and psychological distress, wherein insomnia may act both as a consequence and as a precipitating factor through its neurobiological and behavioral effects. Understanding these interconnections underscores the importance of early intervention in sleep problems to mitigate downstream cardiovascular and cognitive complications.

Current therapeutic approaches vary between Western pharmacological interventions and Chinese herbal medicine. Pharmacotherapy typically includes sedative-hypnotics and menopausal hormone therapy (MHT), which are effective in relieving vasomotor symptoms and preventing osteoporosis. However, long-term MHT use carries risks such as thromboembolic events and breast cancer (5, 6). In contrast, acupuncture as a core modality of (Traditional Chinese Medicine) TCM has garnered increasing attention for its multimodal therapeutic mechanisms. Evidence has already suggested that acupuncture exerts its effects through 3 primary pathways: (1) neuroendocrine regulation via the hypothalamic–pituitary-ovarian (HPO) axis to stabilize hormonal levels (7); (2) neuromodulation, enhancing central neurotransmitters such as serotonin and *γ*-aminobutyric acid (GABA), which contribute to improved mood and sleep quality (8); (3) immunomodulation, reducing inflammatory cytokines like interleukin-6 (IL-6), which are linked to neuroendocrine dysfunction and sleep disturbances (9). Recent advances in neuroimmunology have uncovered striking similarities in the pathophysiological pathways connecting neuroinflammation with concurrent sleep and cognitive disturbances. Studies of anti-CASPR2 encephalitis demonstrate that autoantibody interference with potassium channel complexes induces dual pathology—disrupting both memory consolidation (notably causing retrograde amnesia) and sleep architecture through combined hippocampal damage and inflammatory mediator release (10). This phenomenon bears remarkable resemblance to the cytokine-driven hippocampal sensitization seen in PMI, where elevated IL-6 and other inflammatory markers similarly impair memory networks and sleep–wake regulation. Such cross-condition parallels highlight immunoneuroendocrine pathways as critical therapeutic targets for sleep-cognitive comorbidities.

Currently, integrated AP has emerged as a promising strategy that may enhance therapeutic efficacy while making up the limitations of conventional pharmacological approaches (11). Preclinical studies have suggested that acupuncture may influence neuropharmacological pathways, potentially affecting drug metabolism, receptor activity, and central responsiveness (12). In areas such as chronic pain and addiction, acupuncture has been shown to reduce dependence on opioids, lower required dosages, and minimize adverse drug reactions (13, 14). Although such evidence is primarily drawn from these fields, similar neuromodulatory effects may be relevant to the treatment of hormonal insomnia.

However, pharmacotherapy alone often encounters issues such as tolerance, withdrawal symptoms, and residual sedation (15). Despite increasing interest in integrated approaches, current clinical evidence for AP in treating PMI remains limited. Systematic reviews have identified significant methodological inconsistencies, including heterogeneity in outcome measures, variability in intervention protocols (e.g., frequency, duration, and acupoint selection), and insufficient long-term follow-up. These limitations hamper the development of standardized clinical guidelines.

This study aims to systematically evaluate the efficacy of AP interventions for PMI by synthesizing data from RCTs. By standardizing outcome measures and comparing the effects of various interventions, this meta-analysis aims to provide an evidence-based foundation for integrative treatment strategies in the management of PMS.

## Methods

### Study registration

According to the *PRISMA 2020 statement* (16), we registered the systematic review protocol in PROSPERO on August 20, 2024 (Registration Number: CRD42024579691).^1^

### Search strategies

PubMed, WOS, Cochrane Library, Embase, CNKI, CBM, Wanfang and CQVIP databases were searched, and the search period was set as from the construction of the library to August 1, 2024. The search utilized both MeSH terms and text word. The PubMed search strategy is detailed in Table 1.

**Table 1:** Search strategy (PubMed).

Search	Query
#1	“Acupuncture” [MeSH Terms]
#2	“Acupuncture” [Text Word] OR “Scalp Acupuncture” [Text Word] OR “Neck Acupuncture” [Text Word] OR “Auricular Acupuncture” [Text Word] OR “Facial Acupuncture” [Text Word] OR “Tongue Acupuncture” [Text Word] OR “Hand Acupuncture” [Text Word] OR “Foot Acupuncture” [Text Word] OR “Body Acupuncture” [Text Word] “Abdominal Acupuncture” [Text Word] OR “Back Acupuncture” [Text Word] OR “Wrist-Ankle Acupuncture” [Text Word]
#3	#7 OR #8
#4	“Perimenopause” [MeSH Terms]
#5	“Perimenopause” [Text Word] OR “Menopause” [Text Word] “Menopausal Transition” [Text Word]
#6	#1 OR #2
#7	“Insomnia” [MeSH Terms]
#8	“Insomnia” [Text Word] OR “Sleep Disorder” [Text Word] “Bumei” [Text Word]
#9	#4 OR #5
#10	“Randomized Controlled Trials” [MeSH Terms]
#11	“Randomized Controlled Trials” [Text Word] OR “Randomized Controlled Trial” [Text Word] OR “RCTs” [Text Word] OR “RCT” [Text Word]
#12	#10 OR #11
#13	#3 AND #6 AND #9 AND #12

### Eligibility criteria

Studies were selected based on the pre-defined inclusion criteria: (1) Study design: Only RCTs were considered for inclusion; (2) Participants: Only perimenopausal women who met internationally recognized diagnostic criteria or guidelines for PMI were considered for inclusion, with no restrictions on age, course, ethnicity, country; (3) Interventions: All experimental groups were treated with the AP, while all control groups were treated with Western medication alone (as opposed to the Western medication used in experimental group); (4) Outcome measures: ① Primary outcome measures: Effective rate, HAMA, TCMS; ② Secondary outcome measures: PSQI, KMI, LH, FSH, E_2_; (5) Language restrictions: Only Chinese or English articles were considered for inclusion.

### Study selection

6 researchers (BY, SJ, YT, YW, JZ, CG) were randomly paired into teams of 2 to conduct literature screening using Endnote 20, maintaining independence both between and within groups throughout the screening process. After all groups completed their assessments, inter- and intra-group comparisons were conducted to cross-verify results, identify and reconcile any discrepancies, and ultimately adopt the most comprehensive findings. In case of disagreement, a reviewer (CS) was consulted to reach consensus.

### Data extraction

Six researchers (BY, SJ, YT, YW, JZ, CG) were randomly paired into teams of 2 to conduct literature screening using Excel, maintaining independence both between and within groups throughout the extraction process. A data pre-extraction was conducted first. Upon completion by all groups, inter- and intra-group cross-verification was performed to consolidate the final data extraction results. In case of disagreement, a reviewer (CS) was consulted to reach consensus. Data extraction involved identification (first author and publication date), interventions (experiment group and control group), sample size, age, course, acupuncture points, medication dosages, duration, and outcome measures.

### Quality assessment

Risk of bias of included studies was assessed using the methods recommended by the *Cochrane Handbook for Systematic Reviews of Interventions* (17). The main body consists of 7 items: (1) Random sequence generation (selection bias); (2) Allocation concealment (selection bias); (3) Blinding of participants and personnel (performance bias); (4) Blinding of outcome assessment (detection bias); (5) Incomplete outcome data (attrition bias); (6) Selective reporting (reporting bias); (7) Others (other bias). Each entry was assessed according to the criteria of “low risk,” “unclear risk,” or “high risk.” 6 researchers (BY, SJ, YT, YW, JZ, CG) were randomly paired into teams of 2 to conduct risk-of-bias assessment using RevMan 5.4, maintaining independence both between and within groups throughout the extraction process. Upon completion by all groups, both inter- and intra-group cross-verifications were performed to minimize potential errors. In case of disagreement, a reviewer (CS) was consulted to reach consensus.

### Missing data handling

In cases of missing data, we attempted to contact the original authors for clarification. If unobtainable, the studies were excluded with justification, and sensitivity analyses were conducted to assess potential impacts on the overall findings.

### Statistical methods

The meta-analysis was performed using Review Manager 5.4 and Stata 15.0, employing risk ratios (RR) for dichotomous variables (count data) and mean differences (MD) for continuous variables (measurement data), both reported with 95% confidence intervals (CI). Heterogeneity was evaluated using *I^2^* statistics and *p*-value, with fixed-effect models (*I^2^* ≤ 50%, *p* > 0.1) or random-effects models (*I^2^* > 50%, *p* < 0.1), supplemented by sensitivity, subgroup and regression analyses as needed. For outcome measures (included studies number≥10), funnel plots were constructed and Egger test and Begg test were performed to assess potential publication bias.

### Evidence quality evaluation

Six researchers (BY, SJ, YT, YW, JZ, CG) were randomly paired into teams of 2 to conduct assessment using the GRADEprofiler, maintaining independence both between and within groups throughout the evaluation process. After all groups completed their assessments, inter- and intra-group comparisons were conducted to cross-verify results, identify and reconcile any discrepancies, and ultimately adopt the most comprehensive findings. In case of disagreement, a reviewer (CS) was consulted to reach consensus. Finally, the evidence levels of all outcome measures were categorized into 4 standards: “high,” “moderate,” “low” and “very low.”

## Results

### Literature screening process

**Figure 1:**
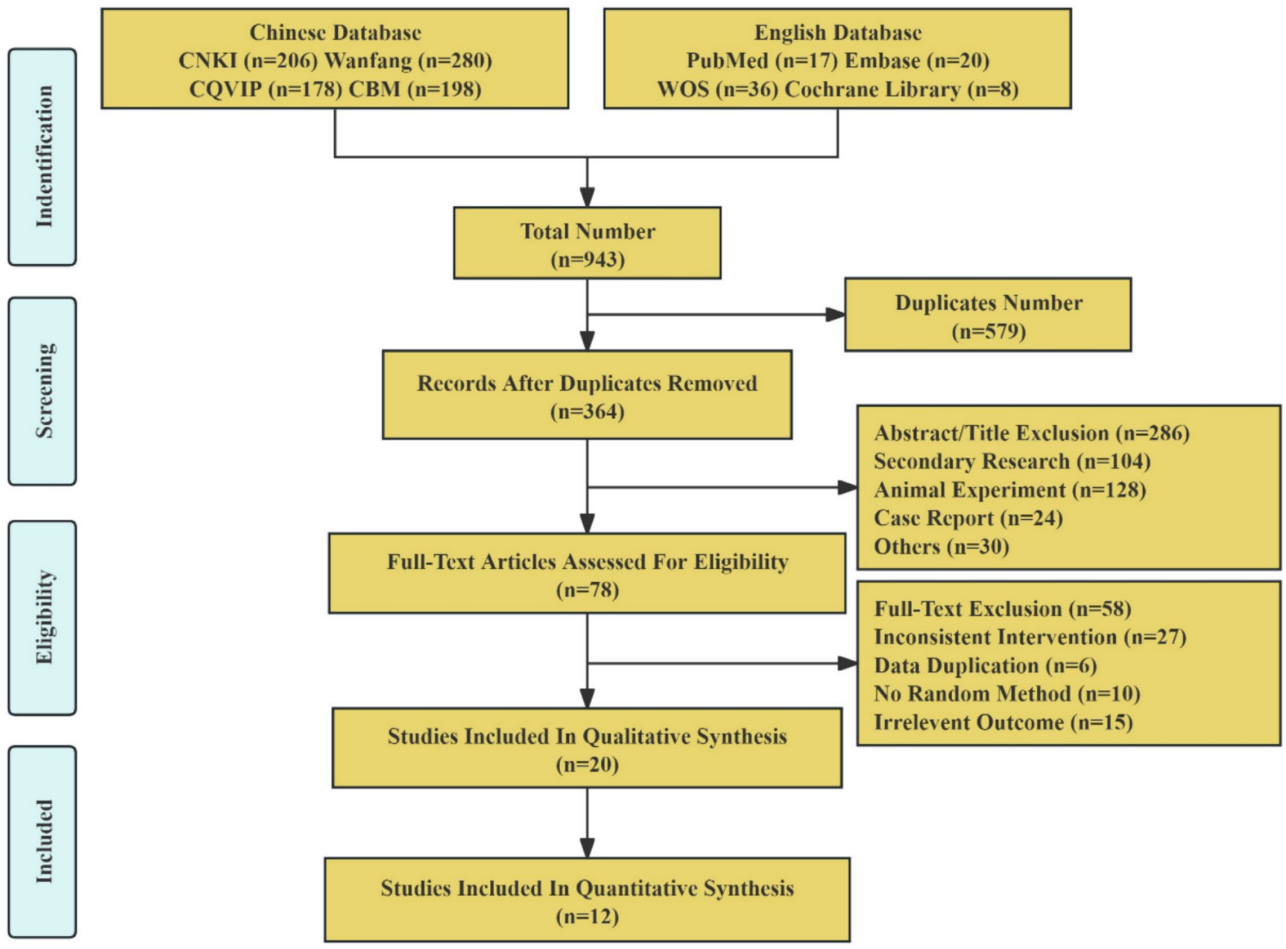
Study flow diagram.

A total of 943 studies underwent initial screening, and through rigorous selection, 12 Chinese studies (18–29) met the eligibility criteria and were included in the final analysis. The literature screening process is detailed in Figure 1.

### Basic characteristics of the studies

The analysis included 12 studies (18–29) comprising 969 PMI patients, with 489 cases in the experiment group and 480 cases in the control group, all showing similar baseline characteristics. The experiment group received AP, while the control group received Western medication alone, with drug regimens corresponding to their respective experiment group. Regarding diagnostic criteria, 4 studies (19, 20, 25, 29) lacked complete standards, including 2 studies (19, 25) that failed to specify perimenopausal criteria and 2 studies (20, 29) that omitted both perimenopausal and insomnia diagnostic criteria. The remaining 8 studies (18, 21–24, 26–28) applied comprehensive criteria, with 4 studies using *CCMD-3* (19, 22, 24, 25) for insomnia diagnosis, 3 studies (19, 21, 22) following TCM Diagnostic & Efficacy Standards, while 5 studies (21–24, 27) utilized Obstetrics and Gynecology for PMI diagnosis. Reported outcome measures included effective rate in 8 studies (18, 20–24, 26, 27), HAMA scores in 4 studies (19, 21, 22, 24), TCMS in 3 studies (21, 23, 28), PSQI in 10 studies (18–25, 27, 29), KMI in 6 studies (19, 21, 23, 24, 26, 28), LH levels in 5 studies (20, 24, 26–28), FSH levels in 6 studies (20, 23, 24, 26–28), and E_2_ levels in 7 studies (20, 23, 24, 26–29). The baseline characteristics of the included studies are summarized in Tables 2–4.

**Table 2:** Basic characteristics of studies.

Study ID	Experimental treatment	Control treatment	Sample size (E/C)	Age [mean ± SD] (E/C)	Course [mean ± SD] (E/C)	Acupuncture points
Bai 2022 (18)	Acu + Zopiclone	Zopiclone	51/51	49.54 ± 1.82/49.11 ± 1.61	2.02 ± 0.81/2.29 ± 0.73 m	BL_23_, HT_7_, SP_6_, Anmian, GV_20_
Dai 2022 (19)	Acu + Escitalopram Oxalate	Escitalopram Oxalate	38/35	48.68 ± 2.28/48.51 ± 3.32	14.34 ± 7.69/13.77 ± 7.24 m	GV_20_, EX-HN_1_, GV_24_, GV_29_
Li 2019 (20)	Acu + Climen	Climen	30/30	55.54 ± 2.94/54.98 ± 2.76	9.71 ± 2.11/9.84 ± 2.43 m	SP_6_, SP_7_, SP_9_
Liu 2023 (21)	Acu + Femoston	Femoston	39/40	49.5 ± 3.0/49.6 ± 2.7	Not mentioned	Hypothalamus, Endocrine, Subcortical, Pituitary, Ovaries, Internal Reproductive Organs (Uterus), Liver, Kidney, Heart, Spleen Sympathetic Nerves, HT_7_, Gonadotropin Point, Body–mind Acupoint, Kuaihuo Point
Zhang 2024 (22)	Acu + Flupentixol-melitracen	Flupentixol-melitracen	35/35	45.69 ± 4.77/44.78 ± 5.23	6.42 ± 3.87/6.26 ± 3.52 m	GV_26_, PC_6_, LR_3_, PC_7_, LI_4_, LI_11_, GB_34_, GB_39_, ST_36_, CV_6_, SP_10_
Zhou 2022 (23)	Acu + Estazolam	Estazolam	35/32	50.37 ± 2.47/49.71 ± 2.71	7.89 ± 2.91/7.94 ± 2.96 m	HT_7_, Heart, Kidney, Sympathetic Nervous System, Endocrine, Subcortical
Xue 2023 (24)	Acu + Estazolam	Estazolam	42/41	48.35 ± 2.37/47.75 ± 3.10	7.34 ± 1.63/8.02 ± 1.46 m	EX-HN_1_, Anmian, GV_20_, BL_62_, LI_4_, ST_40_, LR_14_, LR_2_, LR_3_, BL_18_, KI_6_, SP_6_, ST_36_
Zhu 2016 (25)	Acu + Estazolam	Estazolam	37/37	49.86 ± 3.15/49.27 ± 3.58	2.99 ± 4.24/2.97 ± 3.42 y	GV_20_, GV_24_, EX-HN_1_, Anmian, HT_7_, LR_3_, KI_3_, CV_12_, ST_25_, SP_9_
Lv 2017 (26)	Acu + Nilestriol+Medroxyprogesterone Acetate	Nilestriol+Medroxyprogesterone Acetate	38/38	48.6 ± 3.2/47.7 ± 3.1	2.3 ± 0.3/2.2 ± 0.2 y	GV_20_, BL_15_, BL_20_, BL_23_, BL_18_, BL_13_, CV_4_, CV_3_, HT_7_, SP_6_, ST_36_
Zheng 2023 (27)	Acu + Estazolam+Oryzanol	Estazolam + Oryzanol	45/44	50.38 ± 3.49/50.13 ± 3.26	12.06 ± 3.90/12.68 ± 3.74 m	GV_29_, Anmian, GV_20_, GV_24_, EX-HN_1_, GB_13_, HT_7_, KI_3_, SP_6_
Li 2022 (28)	Acu + Estradiol Valerate + Progesterone	Estradiol Valerate + Progesterone	56/56	51.53 ± 2.02/52.01 ± 2.11	3.28 ± 0.81/3.36 ± 0.78 y	ST_25_, SP_6_, EX-CA_1_, CV_4_
Han 2020 (29)	Acu + Agomelatine	Agomelatine	43/41	Not Mentioned	Not Mentioned	ST_36_, CV_12_, SP_6_, LR_3_, PC_6_

Acu, Acupuncture; E, Experimental group; C, Control group; w, week(s); m, month(s); y, year(s).

**Table 3:** Information supplement.

Study ID	Medication dosages (per dose)	Duration	Outcome measures
Bai 2022 (18)	3 mg	3 months	Effective rate, PSQI
Dai 2022 (19)	5-20 mg	4 weeks	PSQI, HAMA, KMI
Li 2019 (20)	2 mg/1 mg + 2 mg	1 month	Effective rate, PSQI, LH, FSH, E_2_
Liu 2023 (21)	1 mg/1 mg + 10 mg	12 weeks	Effective rate, PSQI, HAMA, KMI, TCMS
Zhang 2024 (22)	0.5 mg + 10 mg	8 weeks	Effective rate, PSQI, HAMA
Zhou 2022 (23)	1 mg	4 weeks	Effective rate, PSQI, KMI, TCMS, FSH, E_2_
Xue 2023 (24)	2 mg	4 weeks	Effective rate, PSQI, HAMA, KMI, LH, FSH, E_2_
Zhu 2016 (25)	1 mg	4 weeks	PSQI
Lv 2017 (26)	1 mg + 2 mg	4 weeks	Effective rate, KMI, LH, FSH, E_2_
Zheng 2023 (27)	1 mg + 10 mg	4 weeks	Effective rate, PSQI, LH, FSH, E_2_
Li 2022 (28)	1 mg + 100 mg	8 weeks	KMI, TCMS, LH, FSH, E_2_
Han 2020 (29)	25 mg	4 weeks	PSQI, E_2_

**Table 4:** Information supplement.

Study ID	Diagnostic criteria (perimenopause)	Diagnostic criteria (insomnia)
Bai 2022 (18)	*International Clinical Practice Guidelines for Traditional Chinese Medicine: Menopausal Syndrome (2020)* *Clinical Application Guidelines of Chinese Patent Medicines for Treating Menopausal Syndrome (2020)*	*Chinese Guidelines for the Diagnosis and Treatment of Adult Insomnia (2017)* *International Clinical Practice Guidelines for Traditional Chinese Medicine: Menopausal Syndrome (2020)* *Clinical Application Guidelines of Chinese Patent Medicines for Treating Menopausal Syndrome (2020)*
Dai 2022 (19)	Not Mentioned	*CCMD-3 (2001)* *TCM Diagnostic & Efficacy Standards (2017)*
Li 2019 (20)	Not Mentioned	Not Mentioned
Liu 2023 (21)	*Clinical Guidelines for Obstetrics and Gynecology (2009)*	*Clinical Guidelines for Obstetrics and Gynecology (2009)* *ICSD-3 (2014)* *TCM Diagnostic & Efficacy Standards (1994)*
Zhang 2024 (22)	*Obstetrics and Gynecology-9 (2018)*	*Obstetrics and Gynecology-9 (2018)* *CCMD-3 (2001)* *TCM Diagnostic & Efficacy Standards (2017)*
Zhou 2022 (23)	*Obstetrics and Gynecology-9 (2018)* *DSM-5 (2018)*	*Obstetrics and Gynecology-9 (2018)* *DSM-5 (2018)* *Guidance principle of clinical study on new drug of traditional Chinese medicine (2002)*
Xue 2023 (24)	*Obstetrics and Gynecology-6 (2004)*	*Obstetrics and Gynecology-6 (2004)* *CCMD-3 (2001)* *Gynecology of Traditional Chinese Medicine-2 (2006)*
Zhu 2016 (25)	Not Mentioned	*Guidelines for Diagnosis and Treatment Programs for 22 Professional and 95 Kinds of Diseases in Chinese Medicine Symptoms (2010)* *Practical Neurology of Integrated Chinese and Western Medicine-2 (2011)* *CCMD-3 (2001)*
Lv 2017 (26)	*Diagnostic Criteria for Gynecological Diseases (2001)*	*Diagnostic Criteria for Gynecological Diseases (2001)*
Zheng 2023 (27)	*Obstetrics and Gynecology-9 (2018)*	*Obstetrics and Gynecology-9 (2018)* *Zhou Zhongying’s Practical Internal Medicine of Traditional Chinese Medicine (2012)*
Li 2022 (28)	*Perimenopausal Syndrome (2011)*	*Perimenopausal Syndrome (2011)*
Han 2020 (29)	Not Mentioned	Not Mentioned

CCMD-3, Chinese Classification of Mental Disorders-3; ICSD-3, International Classification of Sleep Disorders-3; DSM-5, Diagnostic and Statistical Manual of Mental Disorders-5.

### Risk of bias assessment

**Figure 2:**
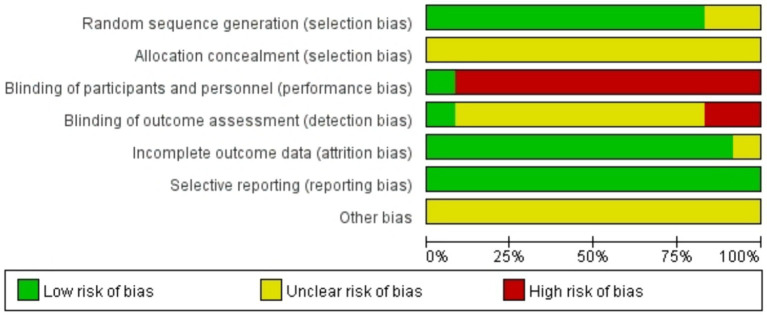
Risk of bias graph.

**Figure 3:**
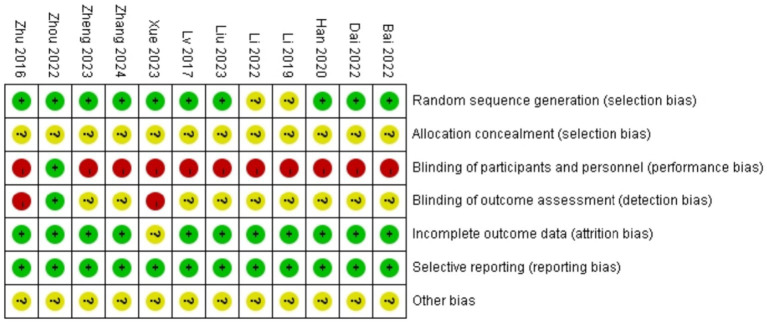
Risk of bias summary.

Among the 12 studies (18–29) included in the analysis, 8 studies (18, 19, 22–27) utilized random number tables for allocation, 2 studies (21, 29) mentioned randomization without specifying methods, and 2 studies (20, 28) employed treatment-based grouping. No studies reported allocation concealment, substantially increasing potential bias risks. Due to the inherent nature of acupuncture interventions, genuine blinding was unattainable. Only 1 study (24) documented blinding of participants and personnel and outcome assessment. Additionally, 1 study had partial missing data that did not significantly affect the analytical outcomes. Detailed results are presented in Figures 2, 3.

### Primary outcome measures

#### Effective rate

**Figure 4:**
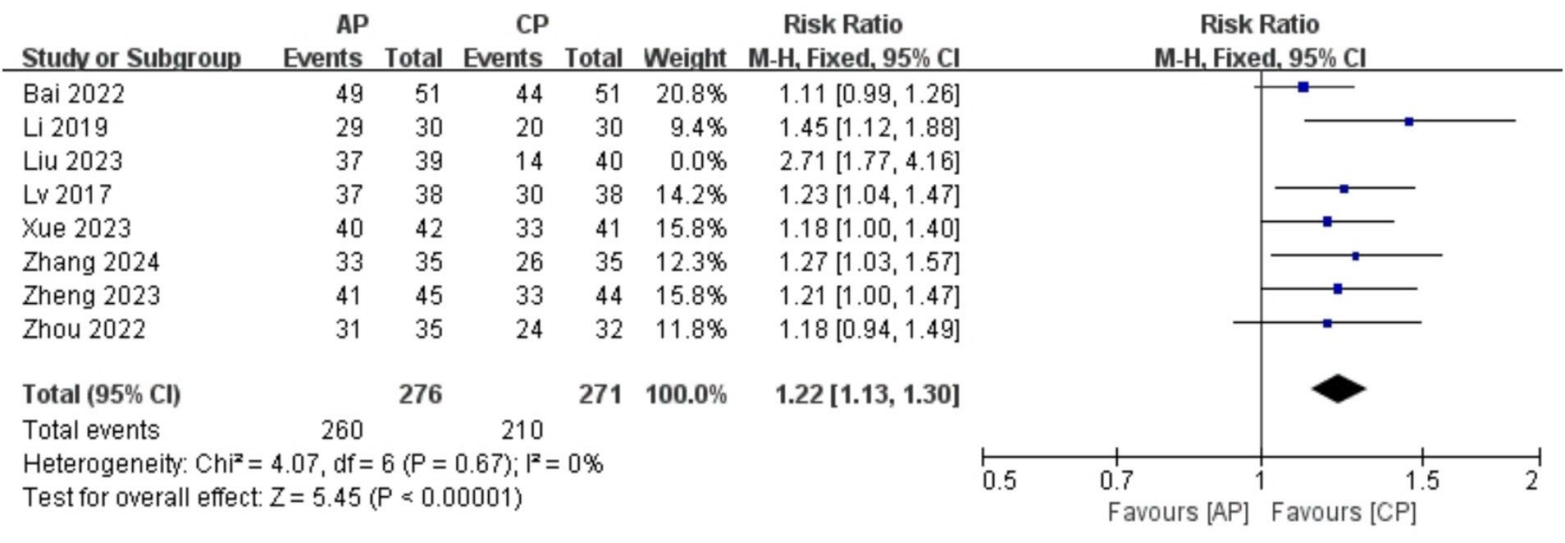
Forest plot effective rate.

A total of 626 patients in 8 studies (18, 20–24, 26, 27) reported the clinical effective rate of AP in PMI. Analysis of the extracted data revealed a moderate level of heterogeneity (*p* = 0.003 < 0.1; *I^2^* = 67%), so a random-effects model was applied [RR = 1.28, 95% CI (1.13, 1.45), *Z* = 3.88, *p* = 0.0001 < 0.05]. Using a stepwise exclusion method, it was found that when the study Liu et al. (21) was excluded, the heterogeneity significantly decreased (*p* = 0.67 > 0.1; *I^2^* = 0%), suggesting that this study may be the source of the heterogeneity. Consequently, a fixed-effects model was applied. Compared with control group, AP effectively improved insomnia symptoms in perimenopausal women, with statistically significant results [RR = 1.22, 95% CI (1.13, 1.30), *Z* = 3.88, *p* < 0.00001], as detailed in Table 5 and Figure 4.

**Table 5:** Sensitivity analysis report of effective rate.

Exclusion	MD [95%CI]	*p*	*I^2^*
Liu 2023 (21)	1.22 [1.13, 1.30]	0.67	0%
Bai 2022 (18)	1.32 [1.15, 1.52]	0.009	65%
Li 2019 (20)	1.26 [1.10, 1.44]	0.003	69%
Xue 2023 (24)	1.31 [1.13, 1.52]	0.001	72%
Lv 2017 (26)	1.30 [1.12, 1.51]	0.001	73%
Zhang 2024 (22)	1.29 [1.12, 1.49]	0.001	73%
Zheng 2023 (27)	1.30 [1.12, 1.51]	0.001	73%
Zhou 2022 (23)	1.30 [1.13, 1.50]	0.001	73%

#### HAMA

**Figure 5:**
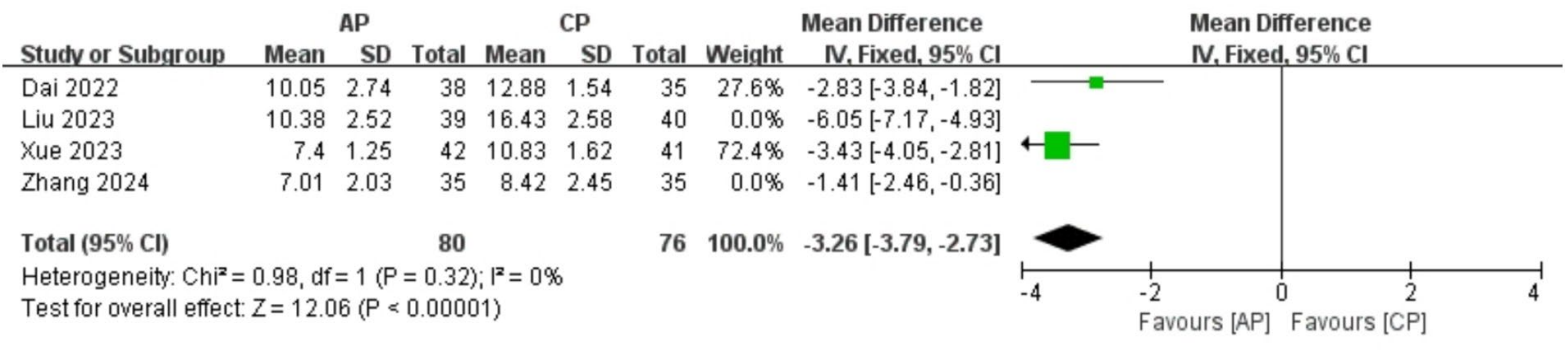
Forest plot of HAMA.

A total of 305 patients in 4 studies (19, 21, 22, 24) reported the HAMA scores of AP in PMI. Analysis of the extracted data indicated high heterogeneity (*p* < 0.00001; *I^2^* = 92%), so a random-effects model was applied [MD = -3.42, 95% CI (−5.03, −1.81), *Z* = 4.16, *p* < 0.00001]. Using a stepwise exclusion method, it was found that when the study Liu et al. (21) and study Zhang et al. (22) were excluded, the heterogeneity significantly decreased (*p* = 0.32 > 0.1; *I^2^* = 0%), suggesting that these 2 studies might be the sources of heterogeneity. Consequently, a fixed-effects model was applied. Compared with control group, AP showed a statistically significant improvement in HAMA scores among perimenopausal women [MD = -3.26, 95% CI (−3.79, −2.73), *Z* = 12.06, *p* < 0.00001], as detailed in Table 6 and Figure 5.

**Table 6:** Sensitivity analysis report of HAMA.

Exclusion	MD [95%CI]	*p*	*I^2^*
Liu 2023 (21)	−2.61 [−3.78, −1.44]	0.005	81%
Zhang 2024 (22)	−4.07 [−5.73, −2.40]	<0.0001	90%
Xue 2023 (24)	−3.42 [−6.03, −0.82]	<0.00001	94%
Dai 2022 (19)	−3.62 [−5.84, −1.40]	<0.00001	94%
Liu 2023 (21) and Zhang 2024 (22)	−3.26 [−3.79, −2.73]	0.32	0%
Liu 2023 (21) and Xue 2023 (24)	−2.13 [−3.52, −0.74]	0.06	72%
Dai 2022 (19) and Liu 2023 (21)	−2.47 [−4.44, −0.49]	0.001	90%
Dai 2022 (19) and Zhang 2024 (22)	−4.70 [−7.26, −2.13]	<0.0001	94%
Xue 2023 (24) and Zhang 2024 (22)	−4.43 [−7.59, −1.27]	<0.0001	94%
Dai 2022 (19) and Xue 2023 (24)	−3.73 [−8.27, −0.82]	<0.00001	97%

#### TCMS

**Figure 6:**
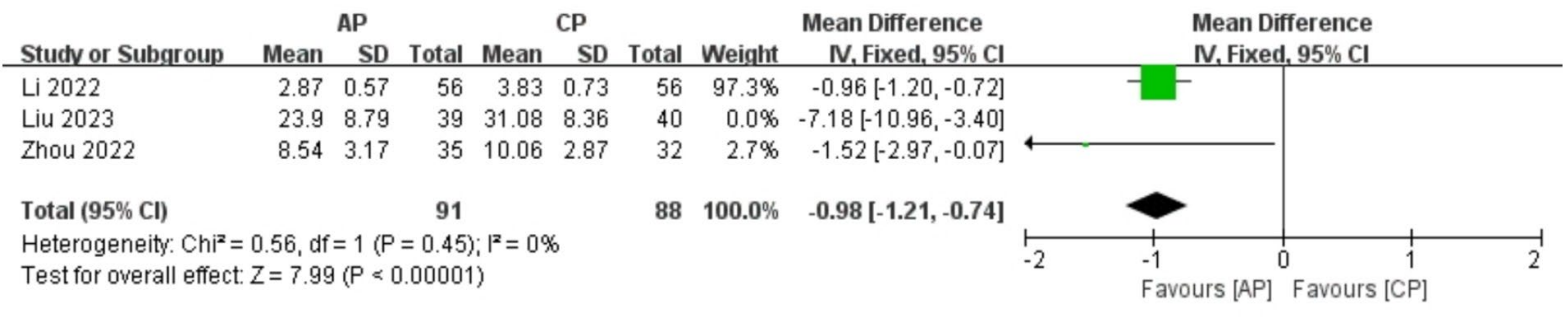
Forest plot of TCMS.

A total of 258 patients in 3 studies (21, 23, 28) reported the TCMS scores of AP in PMI. Analysis of the extracted data indicated high heterogeneity (*p* = 0.004 < 0.1; *I^2^* = 82%), so a random-effects model was applied [MD = −2.22, 95% CI (−4.19, −0.26), *Z* = 2.22, *p* = 0.03 < 0.05]. Using a stepwise exclusion method, it was found that when the study Liu et al. (21) was excluded, the heterogeneity significantly decreased (*p* = 0.45 > 0.1; *I^2^* = 0%), suggesting that this study might be the source of the heterogeneity. Consequently, a fixed-effects model was applied. Compared with control group, AP showed a statistically significant improvement in TCMS scores among perimenopausal women [MD = −0.98, 95% CI (−1.21, −0.74), *Z* = 7.99, *p* < 0.00001], as detailed in Table 7 and Figure 6.

**Table 7:** Sensitivity analysis report of TCMS.

Exclusion	MD [95%CI]	*p*	*I^2^*
Liu 2023 (21)	−0.98 [−1.21, −0.74]	0.45	0%
Li 2022 (28)	−4.07 [−9.59, 1.45]	0.006	87%
Zhou 2022 (23)	−3.77 [−9.84, 2.30]	0.001	90%

### Secondary outcome measures

#### PSQI

**Figure 7:**
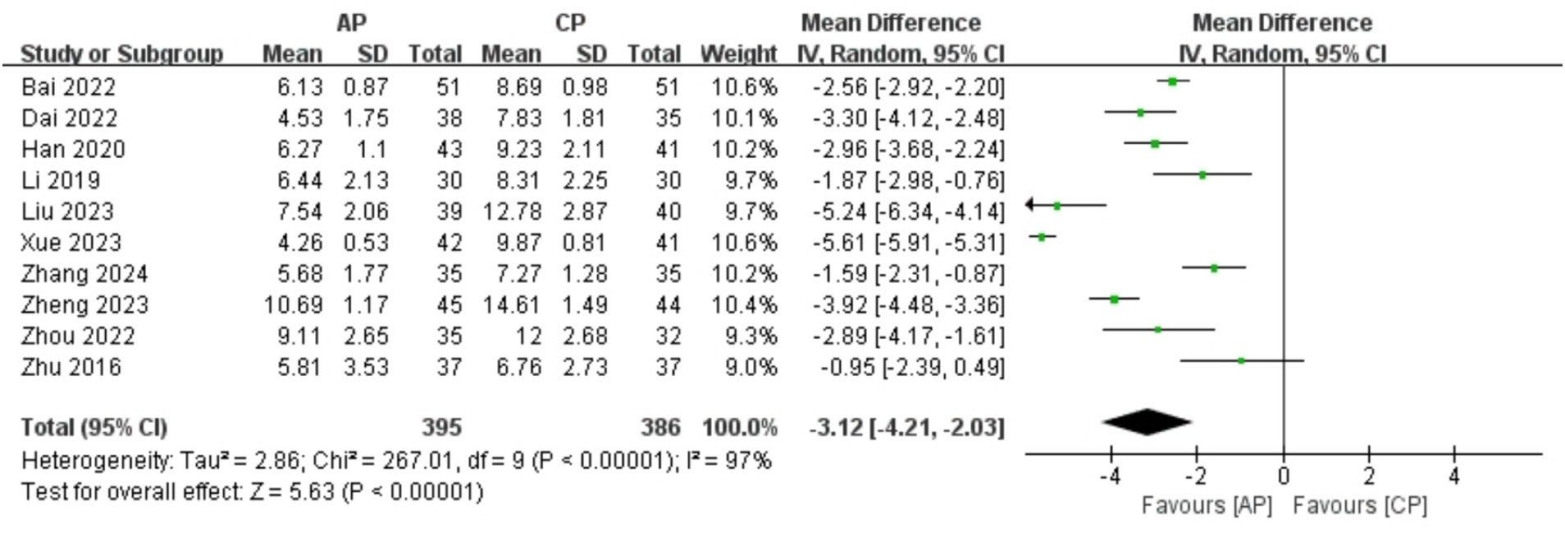
Forest plot of PSQI.

A total of 781 patients in 10 studies (18–25, 27, 29) reported the PSQI of AP in PMI. Analysis of the extracted data indicated high heterogeneity (*p* < 0.00001; *I^2^* = 97%), so a random-effects model was applied. Compared with control group, AP showed a statistically significant improvement in PSQI among perimenopausal women [MD = −3.12, 95% CI (−4.21, −2.03), *Z* = 5.63, *p* < 0.00001]. Using a stepwise exclusion method, no substantial reduction in heterogeneity was observed, and sensitivity analysis confirmed the robustness of the results. Subsequently, subgroup and regression analysis were then performed. The data were categorized into 4 subgroups according to sample size, age, course, and duration, yet the heterogeneity did not significantly decrease. Regression analysis results suggest that although AP may be more effective overall than Western medication alone, an increase in sample size might weaken this effect, with this impact being statistically near significance (*p* = 0.056). The effect sizes for age (*p* = 0.795), course (*p* = 0.466) and duration (*p* = 0.936) were not statistically significant. Regardless of age (<50 years, >50 years), course (<1 year, 1–2 years, 2–3 years) or duration (4 weeks, 8 weeks, 12 weeks), there was little difference in efficacy across subgroups for AP, as detailed in Figure 7 and Table 8.

**Table 8:** Regression analysis results of PSQI.

Covariates	_ES				
	Coef.	Std. Err.	t	*p* > |t|	[95% Conf. Interval]
Sample size	−2.590169	1.160147	−2.23	0.056	−5.265473, 0.0851348
_cons (Sample size)	1.388907	1.711638	0.81	0.441	−2.558137, 5.335952
Age	0.5340293	1.976585	0.27	0.795	−4.139852, 5.207911
_cons (Age)	−2.958072	2.554498	−1.16	0.285	−8.998499, 3.082355
Course	0.9934527	1.276245	0.78	0.466	−2.129406, 4.116311
_cons (Course)	−3.828757	2.121897	−1.80	0.121	−9.020852, 1.363338
Duration	0.075688	0.9084093	0.08	0.936	−2.019108, 2.170484
_cons (Duration)	−2.359632	1.550303	−1.52	0.166	−5.934637, 1.215374

#### KMI

**Figure 8:**
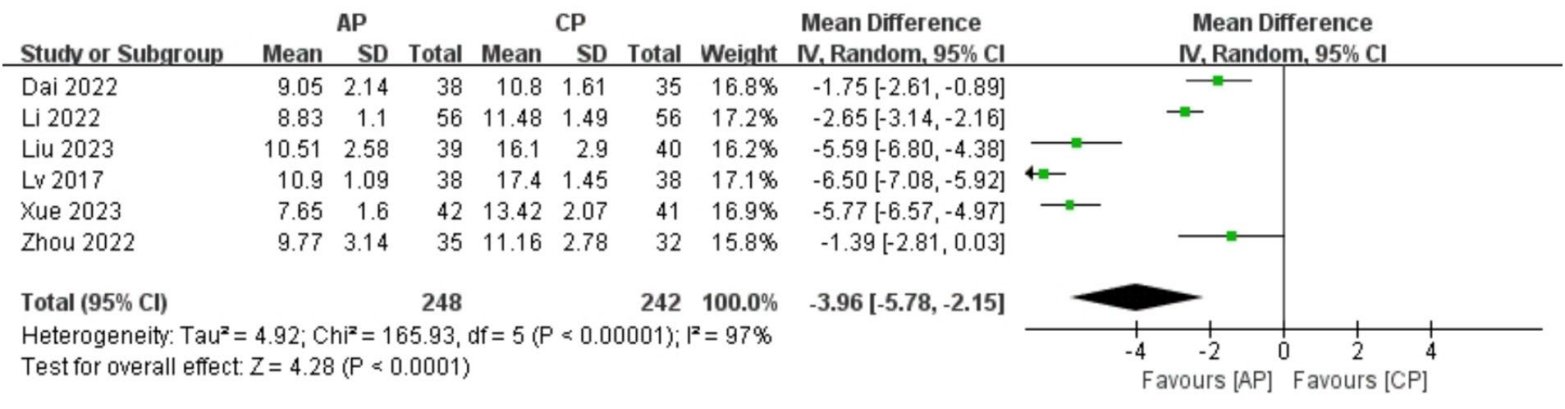
Forest plot of KMI.

A total of 490 patients in 6 studies (19, 21, 23, 24, 26, 28) reported the KMI of AP in PMI. Analysis of the extracted data indicated high heterogeneity (*p* < 0.00001; *I^2^* = 97%), so a random-effects model was applied. Compared with control group, AP showed a statistically significant improvement in KMI among perimenopausal women [MD = −3.96, 95% CI (−5.78, −2.15), *Z* = 4.28, *p* < 0.0001]. Using a stepwise exclusion method, no substantial reduction in heterogeneity was observed, and sensitivity analysis confirmed the robustness of the results. Subsequently, subgroup and regression analysis were then performed. The data were categorized into 4 subgroups according to sample size, age, course, and duration, yet the heterogeneity did not significantly decrease. Regression analysis results showed that the effect sizes for sample size (*p* = 0.774), age (*p* = 0.899), course (*p* = 0.589) and duration (*p* = 0.858) were not statistically significant. Regardless of sample size (<40, >40), age (<50 years, >50 years), course (<1 year, 1–2 years, 2–3 years, >3 years) or duration (4 weeks, 8 weeks, 12 weeks), there was little difference in efficacy between subgroups for AP, as detailed in Figure 8 and Table 9.

**Table 9:** Regression analysis results of KMI.

Covariates	_ES				
	Coef.	Std. Err.	t	*p* > |t|	[95% Conf. Interval]
Sample size	−0.4833773	1.570389	−0.31	0.774	−4.84347,7 3.876723
_cons (Sample size)	−1.602275	2.223611	−0.72	0.511	−7.776009, 4.571458
Age	0.2703314	1.99776	0.14	0.899	−5.27634, 5.817002
_cons (Age)	−2.564183	2.452069	−1.05	0.355	−9.372218, 4.243853
Course	−0.4620522	0.7665194	−0.60	0.589	−2.901459, 1.977354
_cons (Course)	−1.280723	1.907573	−0.67	0.550	−7.351472, 4.790025
Duration	0.1867735	0.9759528	0.19	0.858	−2.522906, 2.896453
_cons (Duration)	−2.529015	1.646977	−1.54	0.199	−7.101756, 2.043726

#### LH

**Figure 9:**
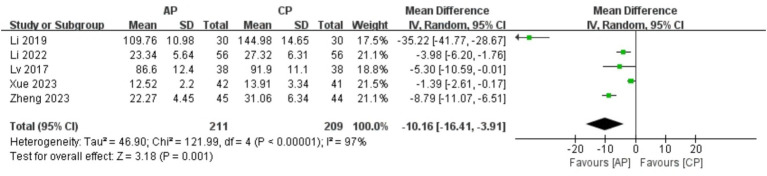
Forest plot of LH.

A total of 420 patients in 5 studies (20, 24, 26–28) reported the LH levels of AP in PMI. Analysis of the extracted data indicated high heterogeneity (*p* < 0.00001; *I^2^* = 97%), so a random-effects model was applied. Compared with control group, AP showed a statistically significant improvement in LH levels among perimenopausal women [MD = −10.16, 95% CI (−16.41, −3.91), *Z* = 3.18, *p* = 0.001 < 0.05]. Using a stepwise exclusion method, no substantial reduction in heterogeneity was observed, and sensitivity analysis confirmed the robustness of the results. Subsequently, subgroup and regression analysis were then performed. The data were categorized into 4 subgroups according to sample size, age, course, and duration, yet the heterogeneity did not significantly decrease. Regression analysis results showed that the effect sizes for sample size (*p* = 0.558), age (*p* = 0.225), course (*p* = 0.390) and duration (*p* = 0.631) were not statistically significant. Regardless of sample size (<40, >40), age (<50 years, >50 years), course (<1 year, 1–2 years, 2–3 years, >3 years) or duration (4 weeks, 8 weeks), there was little difference in efficacy between subgroups for AP, as detailed in Figure 9 and Table 10.

**Table 10:** Regression analysis results of LH.

Covariates	_ES				
	Coef.	Std. Err.	t	*p* > |t|	[95% Conf. Interval]
Sample size	0.6268005	0.9527609	0.66	0.558	−2.40531, 3.658911
_cons (Sample size)	−2.172339	1.602291	−1.36	0.268	−7.271544, 2.926865
Age	−1.14655	0.7530293	−1.52	0.225	−3.543026, 1.249925
_cons (Age)	0.6749386	1.254691	0.54	0.628	−3.318049, 4.667926
Course	0.368096	0.3670972	1.00	0.390	−0.800171, 1.536363
_cons (Course)	−1.978721	0.9226806	−2.14	0.121	−4.915102, 0.9576608
Duration	0.6283968	1.177636	0.53	0.631	−3.119368, 4.376161
_cons (Duration)	−1.921858	1.496743	−1.28	0.289	−6.685162, 2.841446

#### FSH

**Figure 10:**
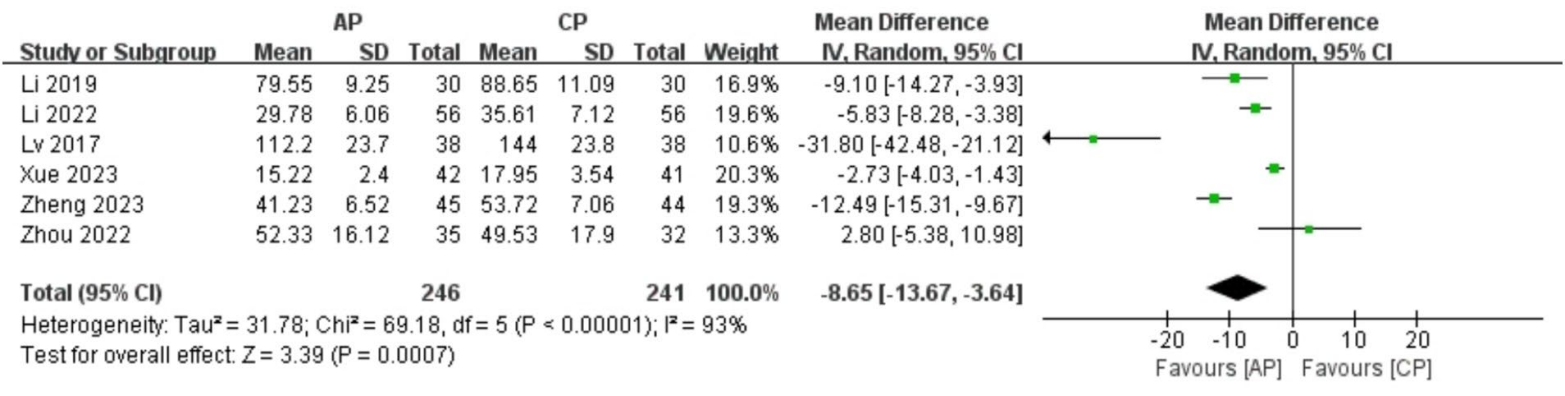
Forest plot of FSH.

A total of 487 patients in 6 studies (20, 23, 24, 26–28) reported the FSH levels of AP in PMI. Analysis of the extracted data indicated high heterogeneity (*p* < 0.00001; *I^2^* = 93%), so a random-effects model was applied. Compared with control group, AP showed a statistically significant improvement in FSH levels among perimenopausal women [MD = −8.65, 95% CI (−13.67, −3.64), *Z* = 3.39, *p* = 0.0007 < 0.05]. Using a stepwise exclusion method, no substantial reduction in heterogeneity was observed, and sensitivity analysis confirmed the robustness of the results. Subsequently, subgroup and regression analysis were then performed. The data were categorized into 4 subgroups according to sample size, age, course, and duration, yet the heterogeneity did not significantly decrease. Regression analysis results showed that the effect sizes for sample size (*p* = 0.398), age (*p* = 0.405), course (*p* = 0.502) and duration (*p* = 0.927) were not statistically significant. Regardless of sample size (<40, >40), age (<50 years, >50 years), course (<1 year, 1–2 years, 2–3 years, >3 years) or duration (4 weeks, 8 weeks), there was little difference in efficacy across subgroups for AP, as detailed in Figure 10 and Table 11.

**Table 11:** Regression analysis results of FSH.

Covariates	_ES_ES				
	Coef.	Std. Err.	t	*p* > |t|	[95% Conf. Interval]
Sample size	−0.515591	0.5448897	−0.95	0.398	−2.028447, 0.9972653
_cons (Sample size)	−0.1685247	0.8656081	−0.19	0.855	−2.571838, 2.234789
Age	−0.5079103	0.5466213	−0.93	0.405	−2.025574, 1.009754
_cons (Age)	−0.1839147	0.8644944	−0.21	0.842	−2.584136, 2.216306
Course	−0.1791234	0.2431741	−0.74	0.502	−0.8542829, 0.496036
_cons (Course)	−0.585141	0.5660585	−1.03	0.360	−2.156771, 0.9864893
Duration	0.0781208	0.7973545	0.10	0.927	−2.13569, 2.291932
_cons (Duration)	−1.038068	0.9826441	−1.06	0.350	−3.766325, 1.690189

#### E_2_

**Figure 11:**
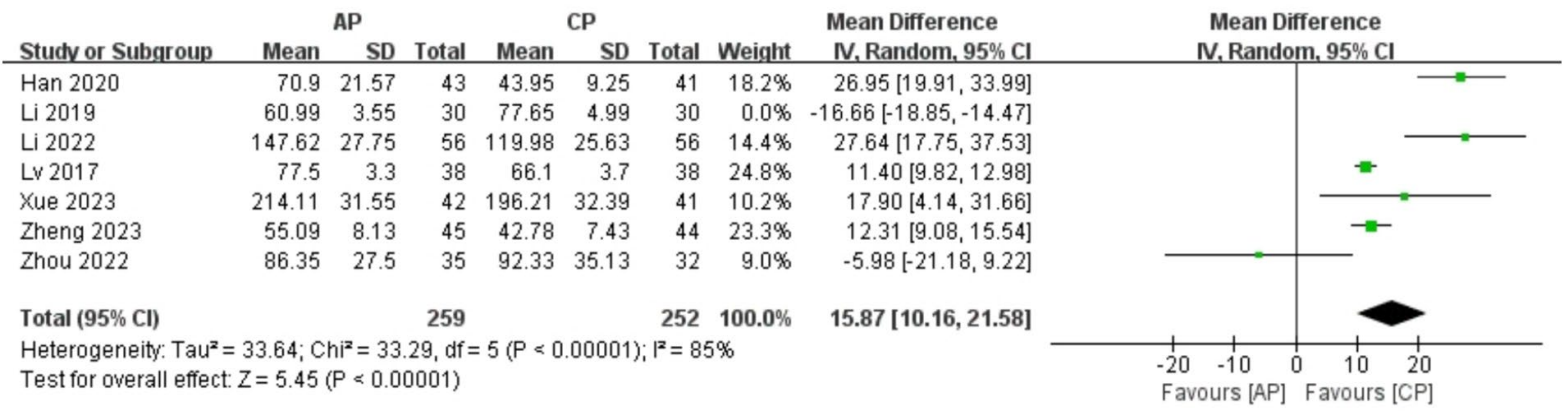
Forest plot of E_2_.

A total of 571 patients in 7 studies (20, 23, 24, 26–29) reported the E_2_ levels of AP in PMI. Analysis of the extracted data indicated high heterogeneity (*p* < 0.00001; *I^2^* = 99%), so a random-effects model was applied. The results showed no significant statistical difference [MD = 10.47, 95% CI (−2.61, 23.56), *Z* = 1.57, *p* = 0.12 > 0.05]. Further investigation revealed that the study Li et al. (20) had a significant impact on the statistical outcome. After excluding this study, the analysis demonstrated statistically significant results (*Z* = 5.45, *p* < 0.00001) along with a reduction in heterogeneity (*p* < 0.00001; *I^2^* = 85%), compared with control group, AP significantly improved E_2_ levels in perimenopausal women [MD = 15.87, 95% CI (10.16, 21.58), *Z* = 5.45, *p* < 0.00001]. Using a stepwise exclusion method to the remaining studies did not substantially reduce heterogeneity, and sensitivity analysis confirmed the robustness of the results. Subgroup and regression analysis were then performed. The data were categorized into 4 subgroups according to sample size, age, course, and duration, yet the heterogeneity did not significantly decrease. Regression analysis results showed that the effect sizes for sample size (*p* = 0.800), age (*p* = 0.935), course (*p* = 0.343) and duration (*p* = 0.835) were not statistically significant. Regardless of sample size (<40, >40), age (<50 years, >50 years), course (<1 year, 1–2 years, 2–3 years, >3 years) or duration (4 weeks, 8 weeks), there was little difference in efficacy across subgroups for AP, as detailed in Figure 11 and Table 12.

**Table 12:** Regression analysis results of E_2_.

Covariates	_ES				
	Coef.	Std. Err.	t	*p* > |t|	[95% Conf. Interval]
Sample size	−0.3025809	1.115643	−0.27	0.800	−3.400103, 2.794941
_cons (Sample size)	1.799774	1.937356	0.93	0.405	−3.579188, 7.178736
Age	0.1198141	1.352561	0.09	0.935	−4.184639, 4.424267
_cons (Age)	1.066501	2.010372	0.53	0.633	−5.331399, 7.464401
Course	0.533321	0.4746068	1.12	0.343	−0.9770897, 2.043732
_cons (Course)	0.0579837	1.181358	0.05	0.964	−3.701624, 3.817592
Duration	−0.312064	1.401123	−0.22	0.835	−4.202205, 3.578078
_cons (Duration)	1.658906	1.7205	0.96	0.390	−3.117968, 6.43578

#### Subgroup analysis, sensitivity analysis and regression analysis

Subgroup analysis was performed based on 4 aspects: sample size (<40, >40), age (<50 years, >50 years), course (<1 year, 1–2 years, 2–3 years, >3 years), and duration (4 weeks, 8 weeks, 12 weeks). See Supplementary material for figures and Tables 13, 14 for details. The analysis revealed that none of the subgroups effectively reduced heterogeneity. Sensitivity analyses showed stable levels, and a stepwise exclusion method was used to identify the sources of heterogeneity. For outcome measures where heterogeneity remained high, regression analysis was performed on each subgroup, as detailed in Tables 8–12 and Supplementary material.

**Table 13:** Subgroup analysis results.

Outcomes			Parameter					
			Studies	Participants	*p*	*I^2^*	Effect estimate	Effect model
PSQI	Sample size	<40	6	423	<0.00001	87%	−2.66 [−3.83, −1.48]	Random
		>40	4	358	<0.0001	98%	−3.77 [−5.46, −2.08]	Random
	Age	<50	7	548	<0.0001	98%	−3.19 [−4.68, −1.70]	Random
		>50	2	149	0.004	90%	−2.95 [−4.96, −0.95]	Random
	Course	<1 years	5	382	0.002	98%	−2.92 [−4.79, −1.06]	Random
		1–2 years	2	162	<0.00001	34%	−3.69 [−4.27, −3.10]	Random
		2–3 years	1	74	0.20	N/A	−0.95 [−2.39, 0.49]	N/A
	Duration	4 weeks	7	530	<0.00001	96%	−3.14 [−4.40, −1.88]	Random
		8 weeks	1	70	<0.0001	N/A	−1.59 [−2.31, −0.87]	N/A
		12 weeks	2	181	0.004	95%	−3.85 [−6.47, −1.22]	Random
KMI	Sample size	<40	4	295	0.007	97%	−3.83 [−6.61, −1.06]	Random
		>40	2	195	0.007	98%	−4.19 [−7.25, −1.14]	Random
	Age	<50	5	378	<0.0001	96%	−4.23 [−6.31, −2.15]	Random
		>50	1	112	<0.00001	N/A	−2.65 [−3.14, −2.16]	N/A
	Course	<1 years	2	150	0.10	96%	−3.62 [−7.91, 0.67]	Random
		1–2 years	1	73	<0.0001	N/A	−1.75 [−2.61, −0.89]	N/A
		2–3 years	1	76	<0.00001	N/A	−6.50 [−7.08, −5.92]	N/A
		>3 years	1	112	<0.00001	N/A	−2.65 [−3.14, −2.16]	N/A
	Duration	4 weeks	4	299	0.002	97%	−3.90 [−6.42, −1.37]	Random
		8 weeks	1	112	<0.00001	N/A	−2.65 [−3.14, −2.16]	N/A
		12 weeks	1	79	<0.00001	N/A	−5.59 [−6.80, −4.38]	N/A
LH	Sample size	<40	2	136	0.18	98%	−20.20 [−49.52, 9.13]	Random
		>40	3	284	0.04	94%	−4.64 [−8.98, −0.31]	Random
	Age	<50	2	159	0.16	50%	−2.46 [−5.88, 0.96]	Random
		>50	3	261	0.006	97%	−15.33 [−26.17, −4.50]	Random
	Course	<1 years	2	143	0.28	99%	−18.15 [−51.30, 15.01]	Random
		1–2 years	1	89	<0.00001	N/A	−8.79 [−11.07, −6.51]	N/A
		2–3 years	1	76	0.05	N/A	−5.30 [−10.59, −0.01]	N/A
		>3 years	1	112	0.0004	N/A	−3.98 [−6.20, −1.76]	N/A
	Duration	4 weeks	4	308	0.008	98%	−12.13 [−21.10, −3.16]	Random
		8 weeks	1	112	0.0004	N/A	−3.98 [−6.20, −1.76]	N/A
FSH	Sample size	<40	3	203	0.13	92%	−12.27 [−28.30, 3.77]	Random
		>40	3	284	0.01	95%	−6.91 [−12.36, −1.45]	Random
	Age	<50	3	226	0.20	93%	−9.85 [−24.76, 5.07]	Random
		>50	3	261	0.0001	84%	−9.10 [−13.77, −4.44]	Random
	Course	<1 years	3	210	0.17	73%	−3.58 [−8.73, 1.56]	Random
		1–2 years	1	89	<0.00001	N/A	−12.49 [−15.31, −9.67]	N/A
		2–3 years	1	76	<0.00001	N/A	−31.80 [−42.48, −21.12]	N/A
		>3 years	1	112	<0.00001	N/A	−5.83 [−8.28, −3.38]	N/A
	Duration	4 weeks	5	375	0.007	94%	−9.73 [−16.78, −2.67]	Random
		8 weeks	1	112	<0.00001	N/A	−5.83 [−8.28, −3.38]	N/A
E_2_	Sample size	<40	3	203	0.75	100%	−3.63 [−26.33, 19.06]	Random
		>40	4	368	<0.0001	85%	20.93 [11.37, 30.49]	Random
	Age	<50	3	226	0.08	66%	8.98 [−1.19, 19.15]	Random
		>50	3	261	0.55	99%	7.42 [−16.98, 31.81]	Random
	Course	<1 years	3	210	0.84	92%	−2.25 [−23.73, 19.22]	Random
		1–2 years	1	89	<0.00001	N/A	12.31 [9.08, 15.54]	N/A
		2–3 years	1	76	<0.00001	N/A	11.40 [9.82, 12.98]	N/A
		>3 years	1	112	<0.00001	N/A	27.64 [17.75, 37.53]	N/A
	Duration	4 weeks	6	459	0.28	99%	7.67 [−6.38, 21.72]	Random
		8 weeks	1	112	<0.00001	N/A	27.64 [17.75, 37.53]	N/A

**Table 14:** Consistency component classification.

Outcomes			Studies	
			Number	ID
PSQI	Sample size	<40	6	Dai 2022 (19), Li 2019 (20), Liu 2023 (21), Zhang 2024 (22), Zhou 2022 (23), and Zhu 2016 (25)
		>40	4	Bai 2022 (18), Xue 2023 (24), Zheng 2023 (27), and Han 2020 (29)
	Age	<50	7	Bai 2022 (18), Dai 2022 (19), Liu 2023 (21), Zhang 2024 (22), Zhou 2022 (23), Xue 2023 (24), and Zhu 2016 (25)
		>50	2	Li 2019 (20) and Zheng 2023 (27)
	Course	<1 years	5	Bai 2022 (18), Li 2019 (20), Zhang 2024 (22), Zhou 2022 (23), and Xue 2023 (24)
		1–2 years	2	Dai 2022 (19) and Zheng 2023 (27)
		2–3 years	1	Zhu 2016 (25)
	Duration	4 weeks	7	Dai 2022 (19), Li 2019 (20), Zhou 2022 (23), Xue 2023 (24), Zhu 2016 (25), Zheng 2023 (27), and Han 2020 (29)
		8 weeks	1	Zhang 2024 (22)
		12 weeks	2	Bai 2022 (18) and Liu 2023 (21)
KMI	Sample size	<40	4	Dai 2022 (19), Liu 2023 (21), Zhou 2022 (23), and Lv 2017 (26)
		>40	2	Xue 2023 (24) and Li 2022 (28)
	Age	<50	5	Dai 2022 (19), Liu 2023 (21), Zhou 2022 (23), Xue 2023 (24), and Lv 2017 (26)
		>50	1	Li 2022 (28)
	Course	<1 years	2	Zhou 2022 (23) and Xue 2023 (24)
		1–2 years	1	Dai 2022 (19)
		2–3 years	1	Lv 2017 (26)
		>3 years	1	Li 2022 (28)
	Duration	4 weeks	4	Dai 2022 (19), Zhou 2022 (23), Xue 2023 (24), and Lv 2017 (26)
		8 weeks	1	Li 2022 (28)
		12 weeks	1	Liu 2023 (21)
LH	Sample size	<40	2	Li 2019 (20) and Lv 2017 (26)
		>40	3	Li 2022 (28), Xue 2023 (24), and Zheng 2023 (27)
	Age	<50	2	Xue 2023 (24) and Lv 2017 (26)
		>50	3	Li 2019 (20), Zheng 2023 (27), and Li 2022 (28)
	Course	<1 years	2	Li 2019 (20) and Xue 2023 (24)
		1–2 years	1	Zheng 2023 (27)
		2–3 years	1	Lv 2017 (26)
		>3 years	1	Li 2022 (28)
	Duration	4 weeks	4	Li 2019 (20), Xue 2023 (24), Lv 2017 (26), and Zheng 2023 (27)
		8 weeks	1	Li 2022 (28)
FSH	Sample size	<40	3	Li 2019 (20), Zhou 2022 (23), and Lv 2017 (26)
		>40	3	Xue 2023 (24), Zheng 2023 (27), and Li 2022 (28)
	Age	<50	3	Zhou 2022 (23), Xue 2023 (24), and Lv 2017 (26)
		>50	3	Li 2019 (20), Zheng 2023 (27), and Li 2022 (28)
	Course	<1 years	3	Li 2019 (20), Zhou 2022 (23), and Xue 2023 (24)
		1–2 years	1	Zheng 2023 (27)
		2–3 years	1	Lv 2017 (26)
		>3 years	1	Li 2022 (28)
	Duration	4 weeks	5	Li 2019 (20), Zhou 2022 (23), Xue 2023 (24), Lv 2017 (26), and Zheng 2023 (27)
		8 weeks	1	Li 2022 (28)
E_2_	Sample size	<40	3	Li 2019 (20), Zhou 2022 (23), and Lv 2017 (26)
		>40	4	Xue 2023 (24), Zheng 2023 (27), Li 2022 (28), and Han 2020 (29)
	Age	<50	3	Zhou 2022 (23), Xue 2023 (24), and Lv 2017 (26)
		>50	3	Li 2019 (20), Zheng 2023 (27), and Li 2022 (28)
	Course	<1 years	3	Li 2019 (20), Zhou 2022 (23), and Xue 2023 (24)
		1–2 years	1	Zheng 2023 (27)
		2−3 years	1	Lv 2017 (26)
		>3 years	1	Li 2022 (28)
	Duration	4 weeks	6	Li 2019 (20), Zhou 2022 (23), Xue 2023 (24), Lv 2017 (26), Zheng 2023 (27), and Han 2020 (29)
		8 weeks	1	Li 2022 (28)

#### Publication bias

**Figure 12:**
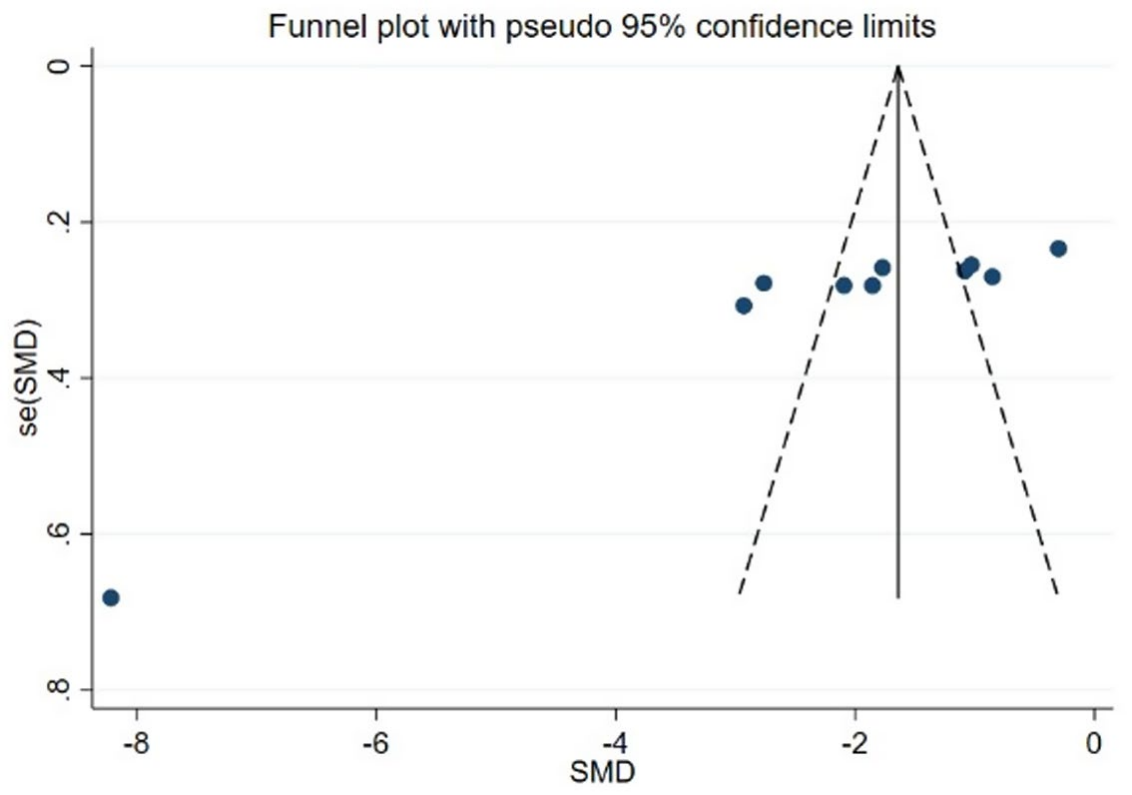
Funnel plot of PSQI.

**Figure 13:**
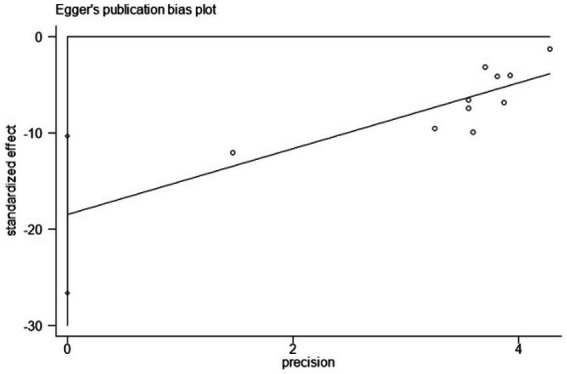
Egger’s test.

**Figure 14:**
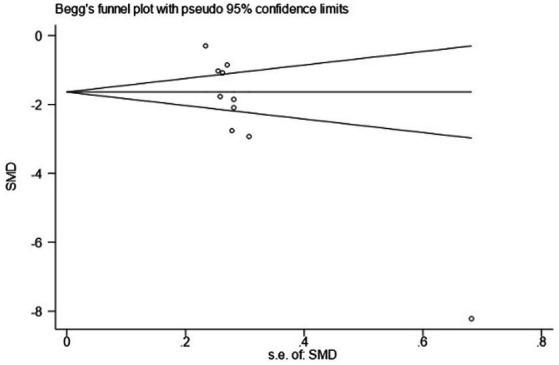
Begg’s test.

Using PSQI (included studies number = 10) as an indicator to draw a funnel plot of reporting bias, the results show that the scatter points are relatively evenly distributed on both sides, but an extreme point is present on the left side, indicating a relatively large standard error, suggesting the possibility of publication bias. The Egger’s test indicates a significantly upward slope, with a slope coefficient of 3.42 (*p* = 0.009) and a bias coefficient of −18.48 (*p* = 0.001). The significant *p*-values suggest a clear presence of publication bias. The Begg’s test further reveals asymmetry in the funnel plot, particularly with several points deviating significantly at the lower end. Kendall’s Score is −31, *z*-value is −2.77, and *p* = 0.006, further supporting the hypothesis of publication bias, as detailed in Figures 12–14 and Tables 15, 16.

**Table 15:** Results of Egger’s test.

Std_Eff	Coef.	Std. Err.	t	*p* > |t|	[95%Conf. Interval]
Slope	3.42073	0.9891879	3.46	0.009	1.139659, 5.701802
bias	−18.48051	3.536957	−5.22	0.001	−26.63674, −10.32427

**Table 16:** Results of Begg’s test.

Outcomes	Results
adj. Kendall’s score (P-Q)	−31
Std. Dev. of Score	11.18
Number of studies	10
*z*	−2.77
Pr > ∣*z*∣	0.006
*z*	2.68 (continuity corrected)
Pr > ∣*z*∣	0.007 (continuity corrected)

#### Grading the quality of evidence

The evidence level assessment indicates that the overall quality of evidence is generally low to very low across all evaluated outcomes (Table 17). This judgment primarily reflects significant methodological limitations present in the included studies. Specifically, the assessment identified serious concerns regarding risk of bias, particularly due to inadequate allocation concealment and insufficient blinding procedures. Furthermore, the certainty of evidence was further diminished by issues of imprecision affecting certain effect estimates.

**Table 17:** Results of the evidence quality assessment.

Profile	Outcomes							
	Effective rate	HAMA	TCMS	PSQI	KMI	LH	FSH	E_2_
Design	RCT	RCT	RCT	RCT	RCT	RCT	RCT	RCT
Studies	8	4	3	10	6	5	6	7
Patients (E/C)	315/311	154/151	130/128	395/386	248/242	211/209	246/241	289/282
Risk of bias	Serious	Serious	Serious	Serious	Serious	Serious	Serious	Serious
Inconsistency	No	No	No	No	No	No	No	No
Indirectness	No	No	No	No	No	No	No	No
Imprecision	No	No	No	Serious	No	Serious	Serious	Very serious
Publication bias	Strongly suspected	Strongly suspected	Strongly suspected	Undetected	Strongly suspected	Strongly suspected	Strongly suspected	Strongly suspected
Other considerations	Reporting bias	Reporting bias	Reporting bias	None	Reporting bias	Reporting bias	Reporting bias	Reporting bias
Relative effect	RR 1.31 (1.21 to 1.41)	None	None	None	None	None	None	None
Absolute effect	223 more per 1,000 (from 151 more to 295 more)	MD 3.42 lower (5.03 to 1.81 lower)	MD 2.22 lower (4.19 to 0.26 lower)	MD 3.12 lower (4.21 to 2.03 lower)	MD 3.96 lower (5.78 to 2.15 lower)	MD 10.16 lower (16.41 to 3.91 lower)	MD 8.65 lower (13.67 to 3.64 lower)	MD 10.47 higher (2.61 lower to 23.56 higher)
	232 more per 1,000 (from 158 more to 307 more)							
Grade	⊕ ⊕ ⊝⊝ Low	⊕ ⊕ ⊝⊝ Low	⊕ ⊕ ⊝⊝ Low	⊕ ⊕ ⊝⊝ Low	⊕ ⊕ ⊝⊝ Low	⊕⊝⊝⊝ Very low	⊕⊝⊝⊝ Very low	⊕⊝⊝⊝ Very low

## Discussion

PMI involve multiple physiological and psychological factors (30, 31). Women in the perimenopausal stage often experience hormonal fluctuations, particularly in estrogen and progesterone, which play a key role in promoting neurotransmitter balance, improving circadian rhythm, adjusting sleep structure, and indirectly influencing mood. When hormonal levels become disrupted, it may lead to sleep disturbances, irritability, and other symptoms (3, 32, 33). Acupuncture modulates the HPO axis by stimulating estrogen receptor (ER)-positive neurons in the hypothalamus, promoting endogenous E_2_ secretion, while downregulating gonadotropin-releasing hormone (GnRH) pulsatility to reduce elevated FSH/LH levels (34). Additionally, acupuncture enhances *β*-endorphin release from the arcuate nucleus, further stabilizing hormonal fluctuations (35, 36). Sleep is not only related to hormonal changes but is also closely linked to autonomic nervous function (37). Due to hormonal fluctuations, the imbalance between the sympathetic and parasympathetic nervous systems results in overactive sympathetic activity at night, leading to issues like rapid heart rate, hot flashes, night sweats, and anxiety, which in turn affect falling asleep and maintaining deep sleep (38). Acupuncture counteracts this by increasing heart rate variability (HRV), reflecting enhanced parasympathetic tone, and reducing nocturnal norepinephrine (NE) release (39, 40). Pharmacological agents like clonidine (an α2-adrenergic agonist) may further suppress sympathetic outflow, but acupuncture provides sustained autonomic nervous system (ANS) rebalancing without drug dependence (41). In TCM, PMS is categorized under conditions like “disorders before and after menopause” and “organ restlessness” associated with both internal and external factors. Clinically, herbal treatments such as Gan Mai Da Zao Decoction for nourishing yin and blood, and Chai Hu Long Gu Mu Li Decoction for relieving depressive fire are commonly used (42, 43). From a biomedical perspective, these formulations may exert effects via anti-inflammatory pathways (downregulating NF-κB and IL-6) and antioxidant activity (enhancing superoxide dismutase [SOD]), which are also targeted by acupuncture (44). Evidence from studies has indicated that acupuncture is effective in managing PMI, and acupuncture combined with Western medication, as an alternative therapy, has shown advantages across multiple outcome measures (45). Acupuncture works by regulating the HPO axis, improving hormone levels in perimenopausal women, significantly reducing LH and FSH levels, and increasing E_2_ levels post-treatment, indicating a positive effect on promoting endogenous hormone secretion and restoring hormonal balance (46). Moreover, acupuncture upregulates serotonin synthesis in the raphe nuclei and GABAergic activity in the hypothalamus, addressing neurotransmitter deficiencies linked to hyperarousal and mood disturbances (47, 48). Acupuncture also shows notable effects in neurological regulation. Research indicates that it can improve anxiety and depression by modulating neurotransmitter levels, such as 5-hydroxytryptamine (5-HT) and dopamine (DA), effectively reducing HAMA scores (49). According to TCM theory, PMI is often caused by liver and kidney yin deficiency or heart and spleen deficiency. Acupuncture enhances TCMS scores by unblocking meridians and harmonizing qi and blood, aligning closely with the holistic concept of TCM (50). Modern studies correlate acupuncture points (GV_20_, HT_7_, SP_6_) with vagal stimulation, 5-HT release, and HPO axis modulation, bridging traditional mechanisms with biomedical evidence (51). The role of Western medication in combination therapy is mainly reflected in its impact on GABA receptors, thereby improving sleep quality, with significant advantages observed in PSQI and KMI improvement (52, 53). AP not only leverages the strengths of both approaches, acupuncture regulates the endocrine system, corrects neurotransmitter imbalances, and alleviates anxiety and depression, while Western medication provides rapid symptom control. The integration of AP produces a synergistic effect, with acupuncture partially mitigating the side effects of Western medication (45), making it a safe alternative therapy.

In this meta-analysis, the majority of included studies employed well-defined diagnostic criteria (mostly internationally recognized standards), while only a minority did not specify their diagnostic methods. This rigorous selection process enhances both the validity and clinical applicability of our findings. By focusing on studies that adopt standardized diagnostic thresholds endorsed by major clinical guidelines, we improved cross-study comparability, minimized diagnostic heterogeneity, and reduced misclassification bias. This methodological consistency ensures that our pooled results are both reliable and generalizable to patient populations meeting these widely accepted criteria. Moreover, the use of clearly defined diagnostic criteria enables more meaningful subgroup analyses and enhances the reproducibility of our study in future research.

Several outcome measures in this meta-analysis exhibited substantial heterogeneity (*I^2^* > 50%), which warrants careful consideration. Potential sources of variability may include differences within patient populations (disease severity, comorbidities), inconsistencies in practitioner technique, and variations in outcome assessment methods (subjective or objective measures) or follow-up durations. Importantly, potential confounding factors such as lifestyle variables (diet, exercise habits), concomitant medication use (hormone therapy, antidepressants), and socioeconomic status were not uniformly reported across studies, which may further contribute to heterogeneity. These factors could independently influence outcomes like sleep quality or mood scores, potentially obscuring the true treatment effect. To address this heterogeneity, we conducted sensitivity analyses by excluding studies with high risk of bias or outliers, which partially reduced inconsistency in some outcomes. Subgroup analyses based on key baseline characteristics (sample size, age, duration, and course) further clarified effect estimates. For outcomes with high heterogeneity, biological mechanisms may offer explanations. For instance, individual variations in hormonal sensitivity (estrogen receptor polymorphisms) or neurotransmitter profiles (serotonin transporter gene variants) could modulate responses to acupuncture or pharmacotherapy. Similarly, variations in sleep outcomes may reflect population differences in how perimenopausal circadian disturbances interact with therapeutic interventions. Nevertheless, residual heterogeneity suggests that unmeasured factors, such as unstandardized co-interventions or publication bias, may still influence results. Given these limitations, the evaluation should be interpreted with caution, particularly for outcomes with high heterogeneity. Future research should prioritize standardized protocols and rigorous reporting to minimize variability and enhance comparability across studies.

Building on established longitudinal methodologies from mental health research, future studies should develop validated clinical prediction tools to identify perimenopausal women most likely to benefit from integrated AP. Three key prognostic domains warrant investigation: (1) biological markers (baseline cortisol, IL-6, and estrogen profiles); (2) sleep architecture parameters (PSQI sub-scores and actigraphy-measured sleep efficiency); (3) psychological phenotypes (HAMA depression cluster scores and stress resilience scales). The proposed framework could adapt linear mixed-effect methods from substance use research to model treatment response trajectories, potentially incorporating dynamic symptom networks mapping insomnia severity to endocrine-immune fluctuations, machine learning analysis of acupoint response patterns from electronic health records, and digital phenotyping via wearable sleep-stage validation —all of which are approaches that would collectively address current evidence gaps in personalized treatment selection for PMI.

Future clinical implementation of acupuncture-pharmacotherapy could benefit from targeted health campaigns and personalized approaches informed by psychological profiles, building on models from vaccination promotion research. Similar to COVID-19 vaccine uptake strategies that considered personality traits and social support, tailored interventions accounting for patients’ stress resilience and health beliefs may optimize treatment adherence. Integration with menopausal health programs could further enhance accessibility and acceptance of this combined therapy.

The strengths of the study are reflected in the following aspects: (1) The study involved a comprehensive search across 8 databases, ensuring a wide scope and thorough content coverage; (2) The analysis included 8 commonly used clinical outcome indicators, making the results more accurate and credible; (3) During the literature inclusion process, strict criteria were applied to select the interventions (with experimental group receiving AP and control group only receiving the corresponding Western medication), which helped to avoid excessive heterogeneity to some extent; (4) The article evaluates the efficacy of AP in PMI, and the analysis results demonstrate that the combination therapy is more advantageous than Western medication alone in treating PMI, highlighting the innovation and unique advantages of TCM combined with pharmacotherapy.

The studies still have some limitations: (1) While our systematic search strategy underwent multiple iterative refinements across 8 databases, the absence of formal peer review by an information specialist represents a potential limitation in search methodology rigor; (2) The limited number of eligible studies, all of which were conducted in Chinese, may lead to potential bias stemming from linguistic or regional influences, and the exclusion of gray literature further restricts the generalizability of findings by omitting potentially relevant unpublished data; (3) When collecting data, the same indicators in different studies had varying units, and some units lack internationally recognized conversion standards, making analysis challenging; (4) Some indicators still showed high heterogeneity, suggesting potential subgroup analyses may be needed; (5) The distinctive characteristics of acupuncture intervention make genuine practitioner blinding methodologically unattainable in clinical research; (6) Currently, high-quality, blinded RCTs are still lacking in clinical practice, which has precluded a comprehensive analysis of the correlation between treatment effects and clinically meaningful thresholds (MCID), and long-term follow-up has also not been achieved; (7) The overall quality of evidence is relatively low; (8) The regression results, while offering exploratory insights, are underpowered due to small subgroup sizes (often <10) and should be viewed as hypothesis-generating given risks of unreliable estimates or spurious associations.

## Conclusion

While the combination therapy of AP demonstrates considerable therapeutic potential, its long-term efficacy and MCID warrant further investigation through large-scale, multicenter RCTs with extended follow-up periods, particularly for distinct insomnia subtypes. Future studies should prioritize protocol optimization to facilitate clinical translation.

## Data Availability

The original contributions presented in the study are included in the article/Supplementary material, further inquiries can be directed to the corresponding author.

## Footnotes

^1^ https://www.crd.york.ac.uk/PROSPERO/view/CRD42024579691
