# Supplementary material for: Integrated acupuncture-pharmacotherapy for perimenopausal insomnia: a systematic review and meta-analysis
Source: Front Neurol. 2025 Aug 20;16:1633794. doi: 10.3389/fneur.2025.1633794 (PMC12406132; doi:10.3389/fneur.2025.1633794)
Supplement: Supplementary file 1 [file Data_Sheet_1.zip › Search Strategy.docx]

| Chinese Database | Search Strategy |
| --- | --- |
| CNKI | #1= (THEME=针刺 + 头针 + 项针 + 耳针 + 面针 + 舌针 + 手针 + 足针 + 体针 + 腹针 + 背针 + 腕踝针 + Acupuncture + Scalp Acupuncture + Neck Acupuncture + Auricular Acupuncture + Facial Acupuncture + Tongue Acupuncture + Hand Acupuncture + Foot Acupuncture + Body Acupuncture + Abdominal Acupuncture + Back Acupuncture + Wrist-Ankle Acupuncture)  #2= (THEME=围绝经期 + 更年期 + 绝经过渡期 + Perimenopause + Menopause + Menopausal Transition)  #3= (THEME=失眠 + 睡眠障碍 + 不寐 + Insomnia + Sleep Disorder + Bumei)  #4= (THEME=随机对照试验 + Randomized Controlled Trials + RCTs + Randomized Controlled Trial + RCT)  #5= #1 * #2 * #3 * #4 (同义词扩展) |
| Wanfang | #1= (THEME=针刺 OR 头针 OR 项针 OR 耳针 OR 面针 OR 舌针 OR 手针 OR 足针 OR 体针 OR 腹针 OR 背针 OR 腕踝针 OR Acupuncture OR Scalp Acupuncture OR Neck Acupuncture OR Auricular Acupuncture OR Facial Acupuncture OR Tongue Acupuncture OR Hand Acupuncture OR Foot Acupuncture OR Body Acupuncture OR Abdominal Acupuncture OR Back Acupuncture OR Wrist-Ankle Acupuncture)  #2= (THEME=围绝经期 OR 更年期 OR 绝经过渡期 OR Perimenopause OR Menopause OR Menopausal Transition)  #3= (THEME=失眠 OR 睡眠障碍 OR 不寐 OR Insomnia OR Sleep Disorder OR Bumei)  #4= (THEME=随机对照试验 OR Randomized Controlled Trials OR RCTs OR Randomized Controlled Trial OR RCT)  #5= #1 AND #2 AND #3 AND #4 (中英文扩展 + 主题词扩展) |
| CQVIP | #1= (THEME=针刺 OR 头针 OR 项针 OR 耳针 OR 面针 OR 舌针 OR 手针 OR 足针 OR 体针 OR 腹针 OR 背针 OR 腕踝针 OR Acupuncture OR Scalp Acupuncture OR Neck Acupuncture OR Auricular Acupuncture OR Facial Acupuncture OR Tongue Acupuncture OR Hand Acupuncture OR Foot Acupuncture OR Body Acupuncture OR Abdominal Acupuncture OR Back Acupuncture OR Wrist-Ankle Acupuncture)  #2= (THEME=围绝经期 OR 更年期 OR 绝经过渡期 OR Perimenopause OR Menopause OR Menopausal Transition)  #3= (THEME=失眠 OR 睡眠障碍 OR 不寐 OR Insomnia OR Sleep Disorder OR Bumei)  #4= (THEME=随机对照试验 OR Randomized Controlled Trials OR RCTs OR Randomized Controlled Trial OR RCT)  #5= #1 AND #2 AND #3 AND #4 (中英文扩展 + 同义词扩展) |
| CBM | #1= (ALL FIELDS=针刺 OR 头针 OR 项针 OR 耳针 OR 面针 OR 舌针 OR 手针 OR 足针 OR 体针 OR 腹针 OR 背针 OR 腕踝针 OR Acupuncture OR Scalp Acupuncture OR Neck Acupuncture OR Auricular Acupuncture OR Facial Acupuncture OR Tongue Acupuncture OR Hand Acupuncture OR Foot Acupuncture OR Body Acupuncture OR Abdominal Acupuncture OR Back Acupuncture OR Wrist-Ankle Acupuncture)  #2= (ALL FIELDS=围绝经期 OR 更年期 OR 绝经过渡期 OR Perimenopause OR Menopause OR Menopausal Transition)  #3= (ALL FIELDS=失眠 OR 睡眠障碍 OR 不寐 OR Insomnia OR Sleep Disorder OR Bumei)  #4= (ALL FIELDS=随机对照试验 OR Randomized Controlled Trials OR RCTs OR Randomized Controlled Trial OR RCT)  #5= #1 AND #2 AND #3 AND #4 |

| English Database | Search Strategy |
| --- | --- |
| PubMed | #1= "Acupuncture" [MeSH Terms]  #2= "Acupuncture" [Text Word] OR "Scalp Acupuncture" [Text Word] OR "Neck Acupuncture" [Text Word] OR "Auricular Acupuncture" [Text Word] OR "Facial Acupuncture" [Text Word] OR "Tongue Acupuncture" [Text Word] OR "Hand Acupuncture" [Text Word] OR "Foot Acupuncture" [Text Word] OR "Body Acupuncture" [Text Word] "Abdominal Acupuncture" [Text Word] OR "Back Acupuncture" [Text Word] OR "Wrist-Ankle Acupuncture" [Text Word]  #3= #1 OR #2  #4= "Perimenopause" [MeSH Terms]  #5= "Perimenopause" [Text Word] OR "Menopause" [Text Word] "Menopausal Transition" [Text Word]  #6= #4 OR #5  #7= "Insomnia" [MeSH Terms]  #8= "Insomnia" [Text Word] OR "Sleep Disorder" [Text Word] "Bumei" [Text Word]  #9= #7 OR #8  #10= "Randomized Controlled Trials" [MeSH Terms]  #11= "Randomized Controlled Trials" [Text Word] OR "Randomized Controlled Trial" [Text Word] OR "RCTs" [Text Word] OR "RCT" [Text Word]  #12= #10 OR #11  #13= #3 AND #6 AND #9 AND #12 |
| WOS | #1: TS= (Acupuncture) OR TS= (Scalp Acupuncture) OR TS= (Neck Acupuncture) OR TS= (Auricular Acupuncture) OR TS= (Facial Acupuncture) OR TS= (Tongue Acupuncture) OR TS= (Hand Acupuncture) OR TS= (Foot Acupuncture) OR TS= (Body Acupuncture) OR TS= (Abdominal Acupuncture) OR TS= (Back Acupuncture) OR TS= (Wrist-Ankle Acupuncture)  #2: TS= (Perimenopause) OR TS= (Menopause) OR TS= (Menopausal Transition)  #3: TS= (Insomnia) OR TS= (Sleep Disorder) OR TS= (Bumei)  #4: TS= (Randomized Controlled Trials) OR TS= (Randomized Controlled Trial) OR TS= (RCTs) OR TS=(RCT)  #5: #1 AND #2 AND #3 AND #4 |
| Embase | #1. 'Acupuncture'/exp  #2. 'Acupuncture'free text OR 'Scalp Acupuncture'free text OR 'Neck Acupuncture'free text OR 'Auricular Acupuncture'free text OR 'Facial Acupuncture'free text OR 'Tongue Acupuncture'free text OR 'Hand Acupuncture'free text OR 'Foot Acupuncture'free text OR 'Body Acupuncture'free text OR 'Abdominal Acupuncture'free text OR 'Back Acupuncture'free text OR 'Wrist-Ankle Acupuncture'free text  #3. #1 OR #2  #4. 'Perimenopause'/exp  #5. 'Perimenopause'free text OR 'Menopause'free text OR 'Menopausal Transition'free text  #6. #4 OR #5  #7. 'Insomnia'/exp  #8. 'Insomnia'free text OR 'Sleep Disorder'free text OR 'Bumei'free text  #9. #7 OR #8  #10. 'Randomized Controlled Trials'/exp  #11. 'Randomized Controlled Trials'free text OR 'Randomized Controlled Trial'free text OR 'RCTs'free text OR 'RCT'free text  #12. #10 OR #11  #13. #3 AND #6 AND #9 AND #12 |
| Cochrane Library | #1 Acupuncture[MeSH descriptor]  #2 Acupuncture[All Text] OR Scalp Acupuncture[All Text] OR Neck Acupuncture[All Text] OR Auricular Acupuncture[All Text] OR Facial Acupuncture[All Text] OR Tongue Acupuncture[All Text] OR Hand Acupuncture[All Text] OR Foot Acupuncture[All Text] OR Body Acupuncture[All Text] OR Abdominal Acupuncture[All Text] OR Back Acupuncture[All Text] OR Wrist-Ankle Acupuncture[All Text]  #3 #1 OR #2  #4 Perimenopause[MeSH descriptor]  #5 Perimenopause[All Text] OR Menopause[All Text] OR Menopausal Transition[All Text]  #6 #4 OR #5  #7 Sleep Initiation and Maintenance Disorders[MeSH descriptor]  #8 Sleep Initiation and Maintenance Disorders[All Text] OR Insomnia[All Text] OR Sleep Disorder[All Text] OR Bumei[All Text]  #9 #7 OR #8  #10 Randomized Controlled Trial[MeSH descriptor]  #11 Randomized Controlled Trial[All Text] OR Randomized Controlled Trials[All Text] OR RCT[All Text] OR RCTs[All Text]  #12 #10 OR #11  #13 #3 AND #6 AND #9 AND #12 |
