# Supplementary figures and images for: Integrated acupuncture-pharmacotherapy for perimenopausal insomnia: a systematic review and meta-analysis
Source: Front Neurol. 2025 Aug 20;16:1633794. doi: 10.3389/fneur.2025.1633794 (PMC12406132; doi:10.3389/fneur.2025.1633794)

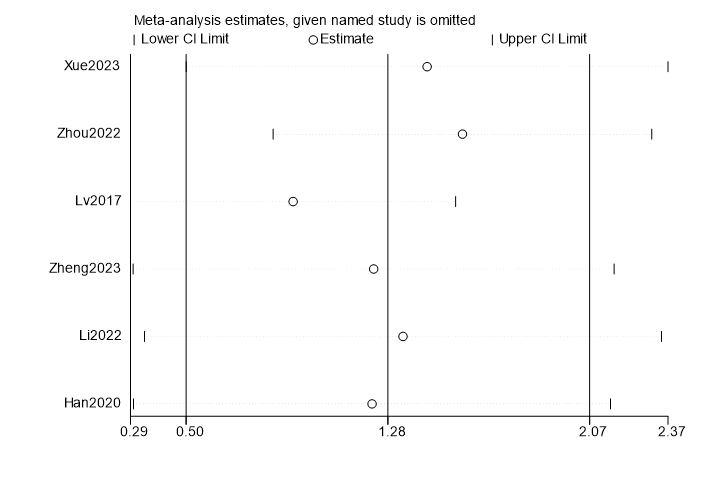

Supplement: Supplementary file 1 [file Data_Sheet_1.zip › Sensitivity analysis/Sensitivity analysis of E2.tif]

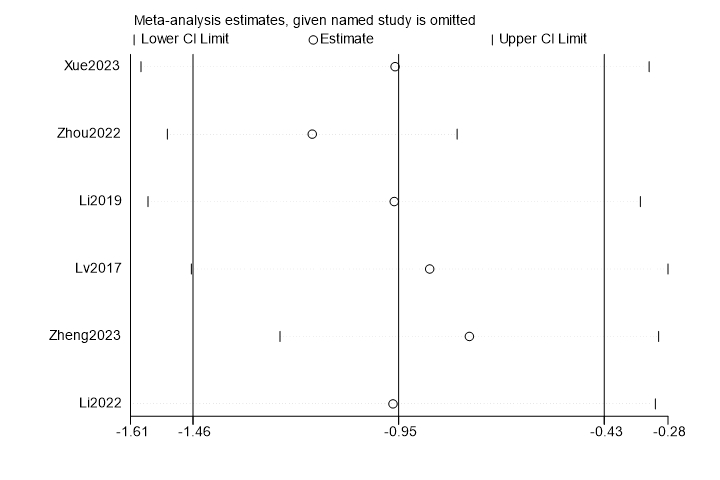

Supplement: Supplementary file 1 [file Data_Sheet_1.zip › Sensitivity analysis/Sensitivity analysis of FSH.tif]

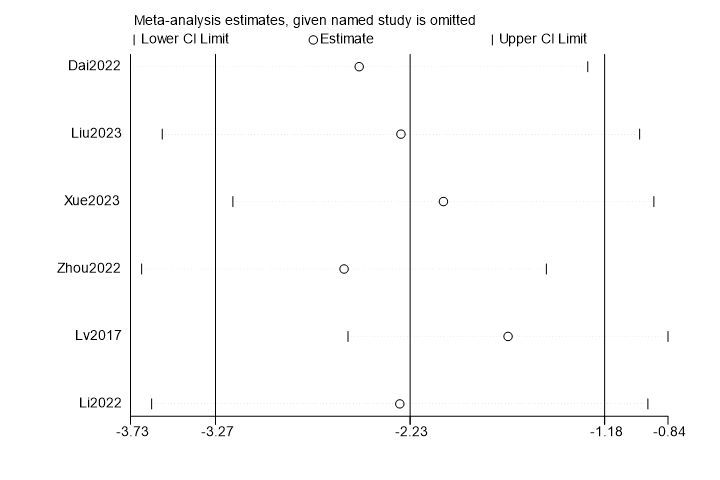

Supplement: Supplementary file 1 [file Data_Sheet_1.zip › Sensitivity analysis/Sensitivity analysis of KMI.tif]

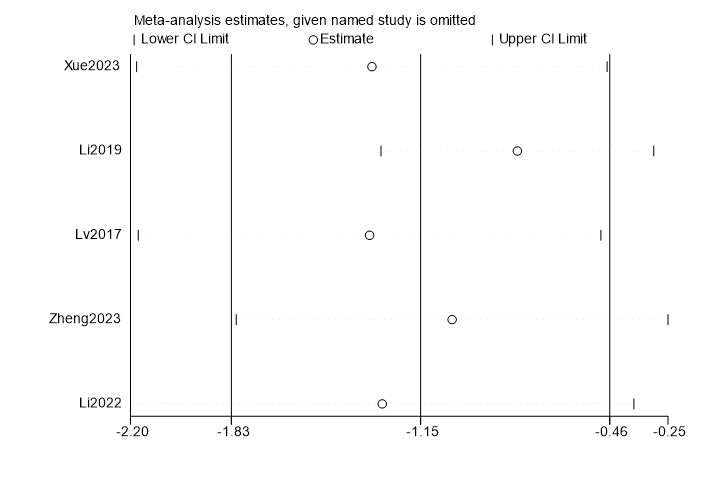

Supplement: Supplementary file 1 [file Data_Sheet_1.zip › Sensitivity analysis/Sensitivity analysis of LH.tif]

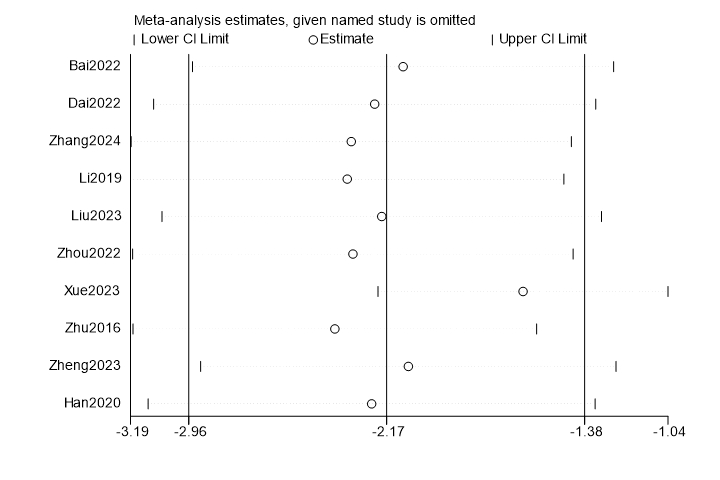

Supplement: Supplementary file 1 [file Data_Sheet_1.zip › Sensitivity analysis/Sensitivity analysis of PSQI.tif]

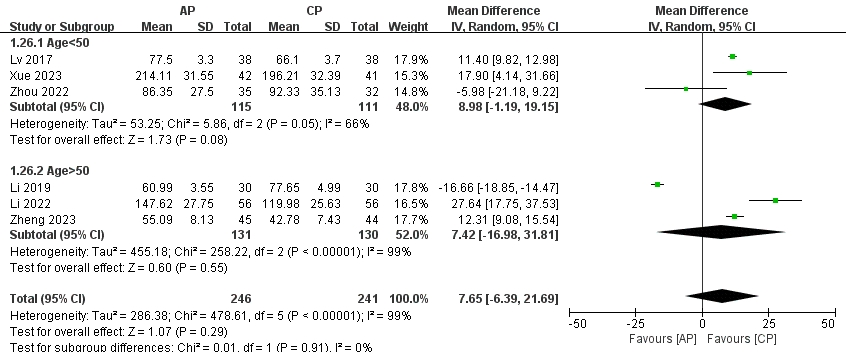

Supplement: Supplementary file 1 [file Data_Sheet_1.zip › Subgroup analysis/Subgroup analysis of E2/Age Subgroup of E2.tif]

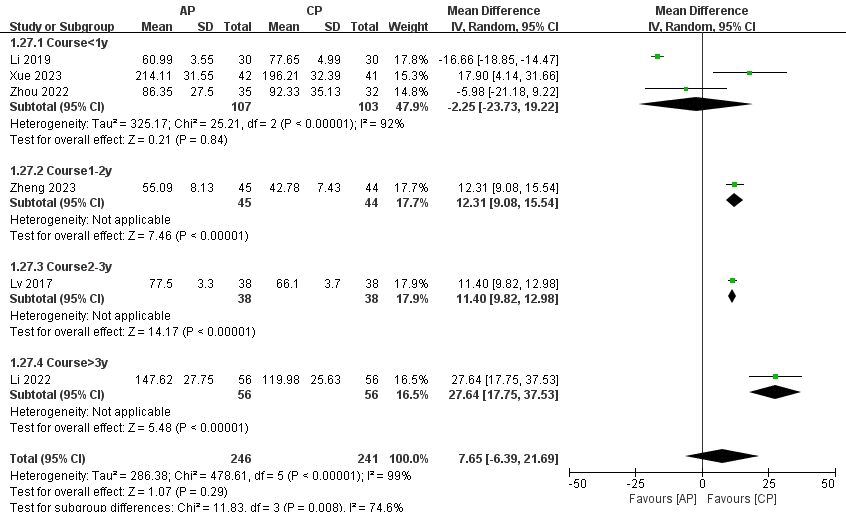

Supplement: Supplementary file 1 [file Data_Sheet_1.zip › Subgroup analysis/Subgroup analysis of E2/Course Subgroup of E2.tif]

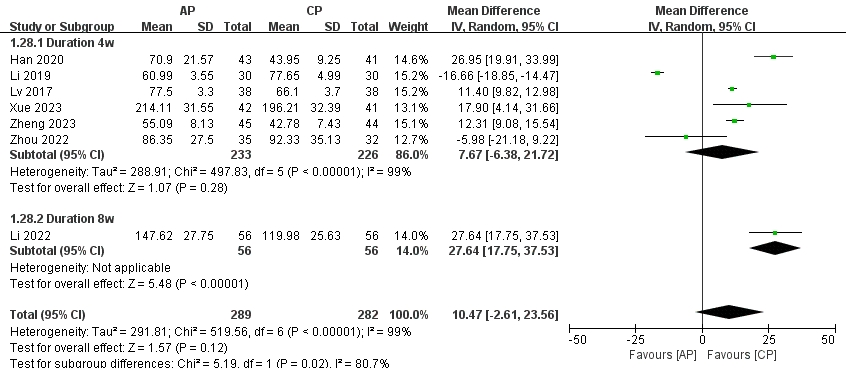

Supplement: Supplementary file 1 [file Data_Sheet_1.zip › Subgroup analysis/Subgroup analysis of E2/Duration Subgroup of E2.tif]

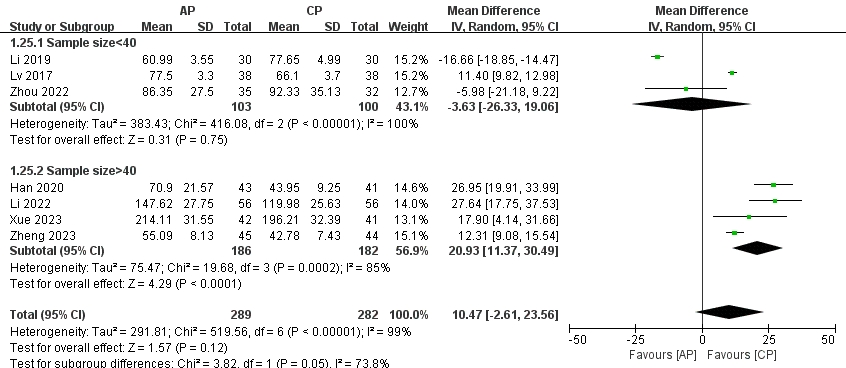

Supplement: Supplementary file 1 [file Data_Sheet_1.zip › Subgroup analysis/Subgroup analysis of E2/Sample size Subgroup of E2.tif]

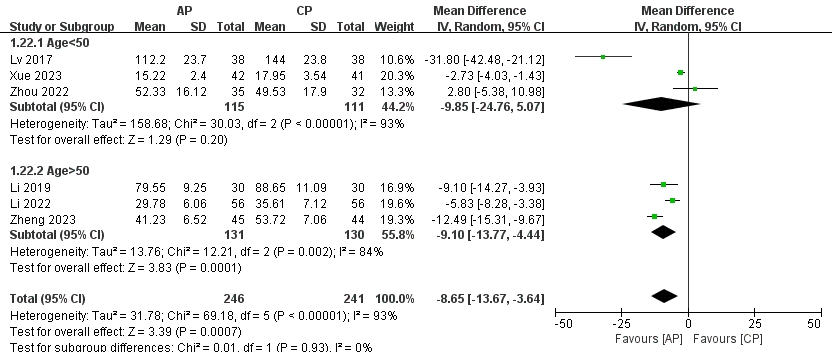

Supplement: Supplementary file 1 [file Data_Sheet_1.zip › Subgroup analysis/Subgroup analysis of FSH/Age Subgroup of FSH.tif]

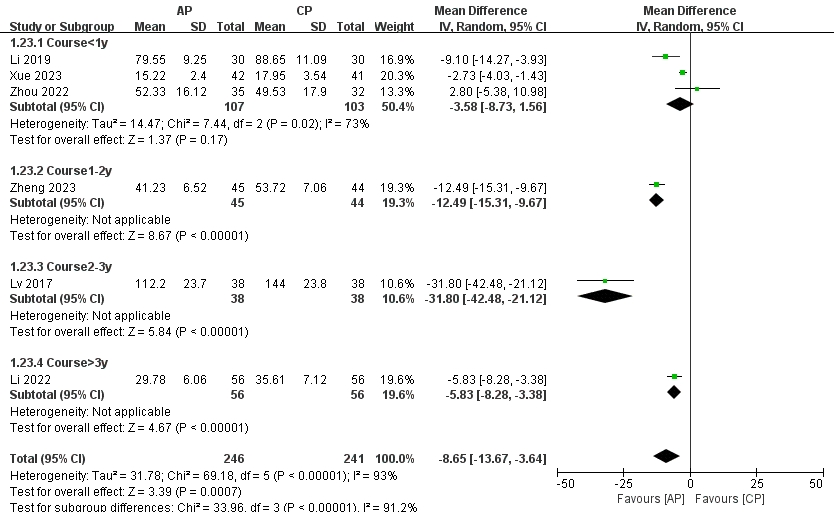

Supplement: Supplementary file 1 [file Data_Sheet_1.zip › Subgroup analysis/Subgroup analysis of FSH/Course Subgroup of FSH.tif]

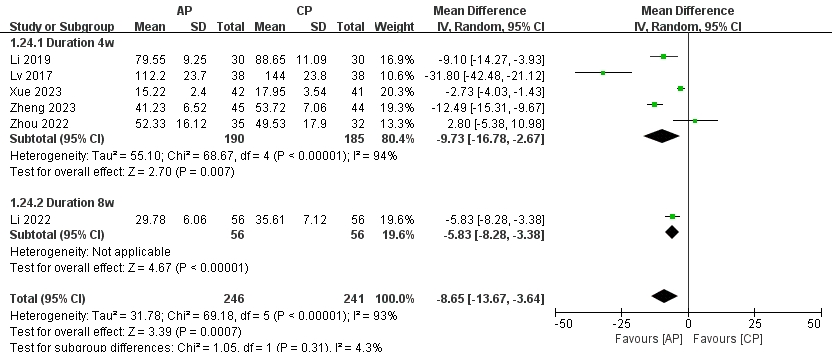

Supplement: Supplementary file 1 [file Data_Sheet_1.zip › Subgroup analysis/Subgroup analysis of FSH/Duration Subgroup of FSH.tif]

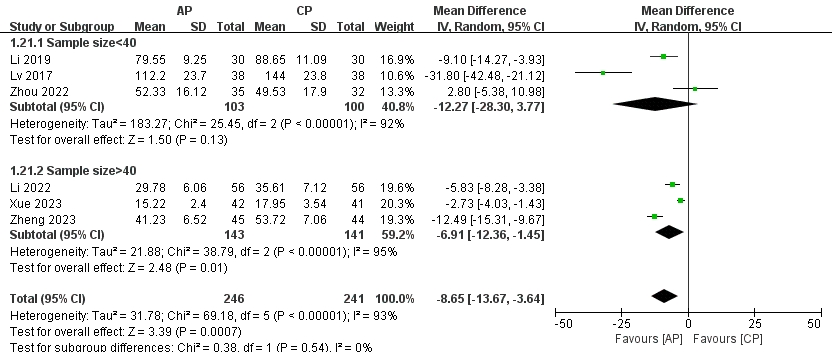

Supplement: Supplementary file 1 [file Data_Sheet_1.zip › Subgroup analysis/Subgroup analysis of FSH/Sample size Subgroup of FSH.tif]

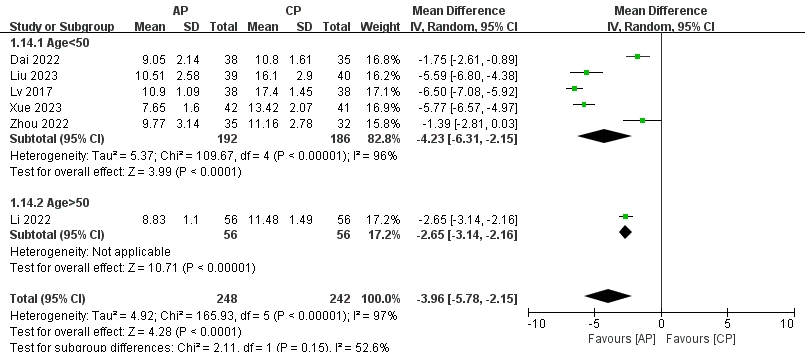

Supplement: Supplementary file 1 [file Data_Sheet_1.zip › Subgroup analysis/Subgroup analysis of KMI/Age Subgroup of KMI.tif]

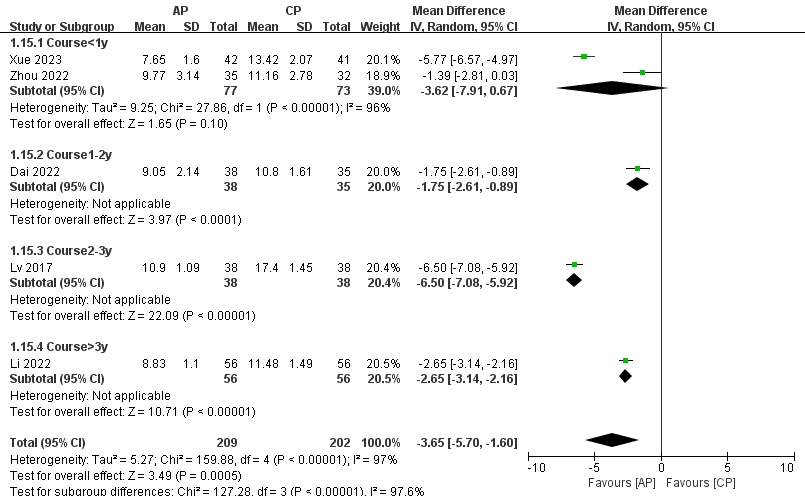

Supplement: Supplementary file 1 [file Data_Sheet_1.zip › Subgroup analysis/Subgroup analysis of KMI/Course Subgroup of KMI.tif]

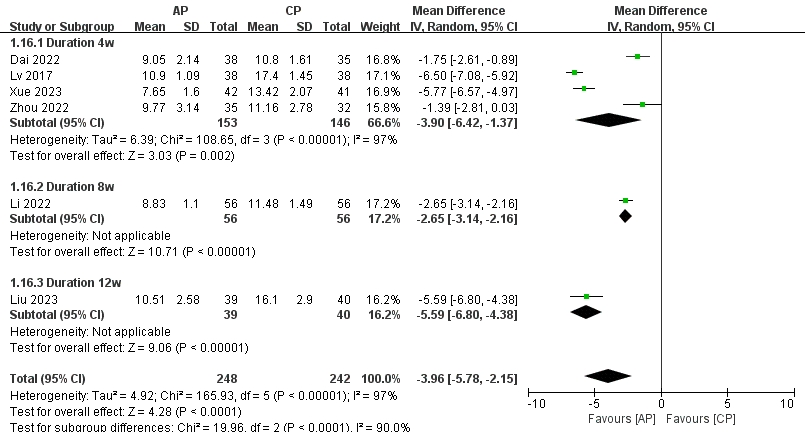

Supplement: Supplementary file 1 [file Data_Sheet_1.zip › Subgroup analysis/Subgroup analysis of KMI/Duration Subgroup of KMI.tif]

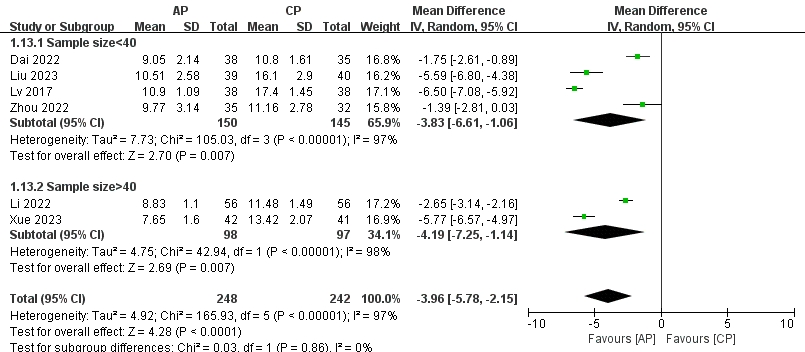

Supplement: Supplementary file 1 [file Data_Sheet_1.zip › Subgroup analysis/Subgroup analysis of KMI/Sample size Subgroup of KMI.tif]

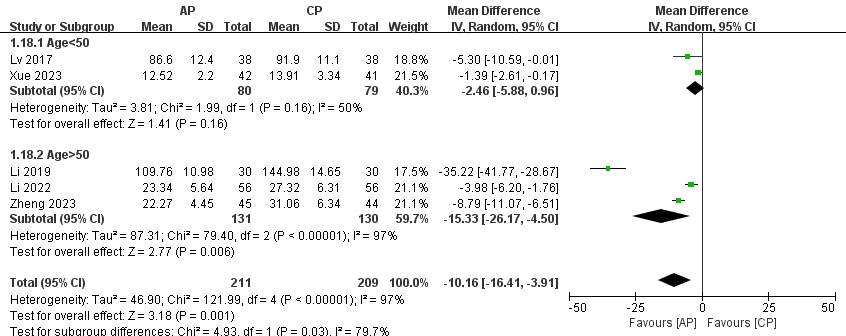

Supplement: Supplementary file 1 [file Data_Sheet_1.zip › Subgroup analysis/Subgroup analysis of LH/Age Subgroup of LH.tif]

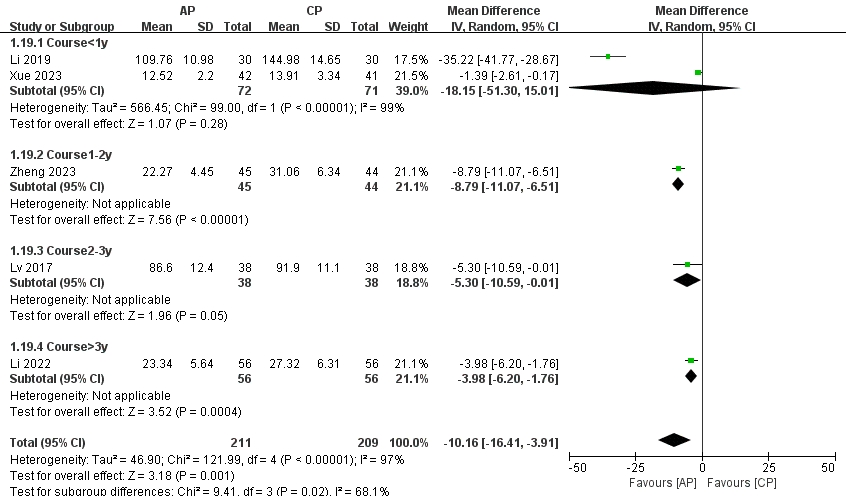

Supplement: Supplementary file 1 [file Data_Sheet_1.zip › Subgroup analysis/Subgroup analysis of LH/Course Subgroup of LH.tif]

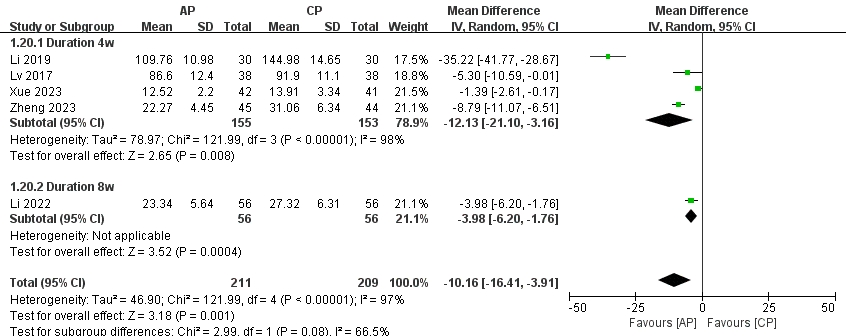

Supplement: Supplementary file 1 [file Data_Sheet_1.zip › Subgroup analysis/Subgroup analysis of LH/Duration Subgroup of LH.tif]

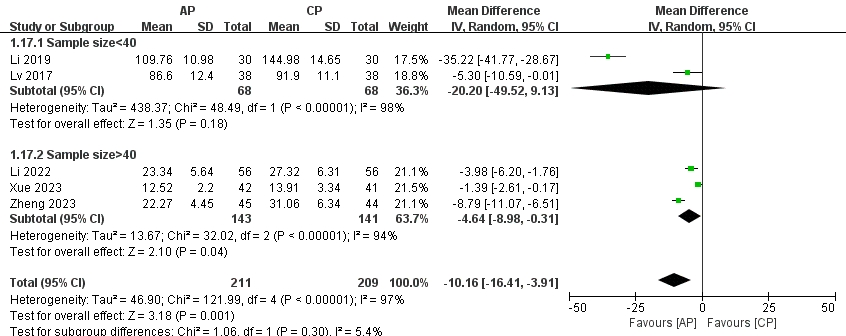

Supplement: Supplementary file 1 [file Data_Sheet_1.zip › Subgroup analysis/Subgroup analysis of LH/Sample size Subgroup of LH.tif]

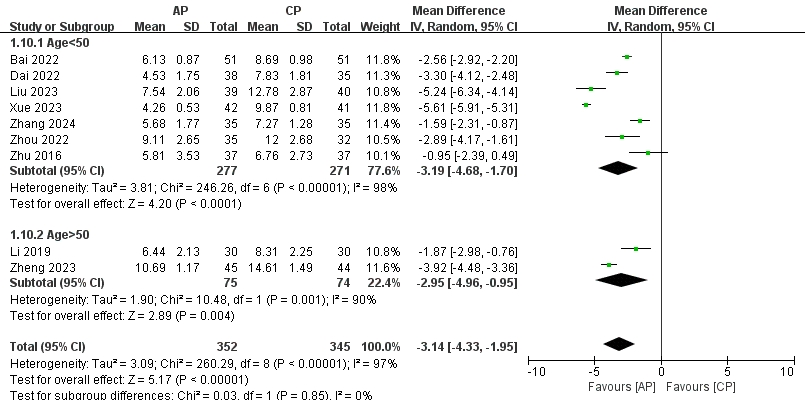

Supplement: Supplementary file 1 [file Data_Sheet_1.zip › Subgroup analysis/Subgroup analysis of PSQI/Age Subgroup of PSQI.tif]

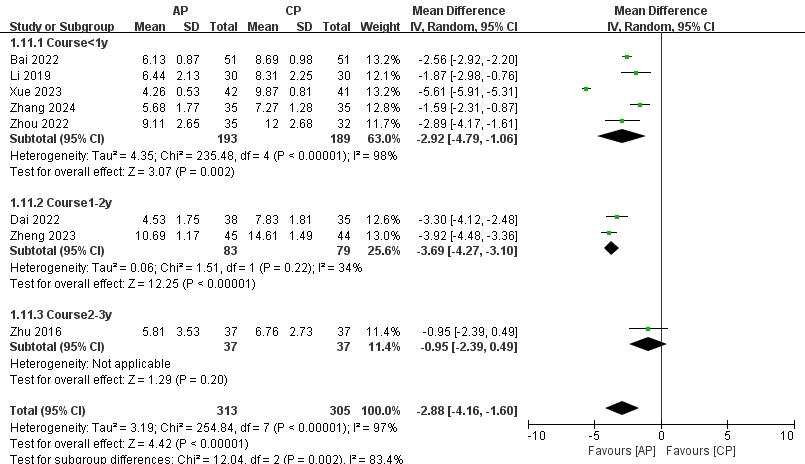

Supplement: Supplementary file 1 [file Data_Sheet_1.zip › Subgroup analysis/Subgroup analysis of PSQI/Course Subgroup of PSQI.tif]

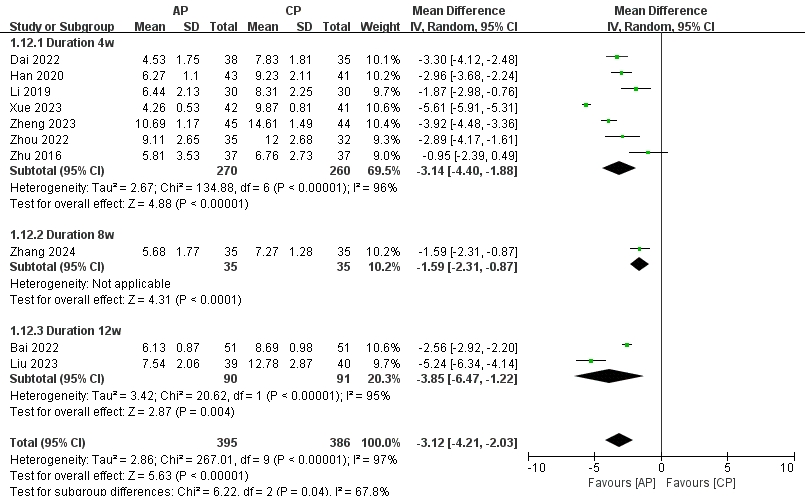

Supplement: Supplementary file 1 [file Data_Sheet_1.zip › Subgroup analysis/Subgroup analysis of PSQI/Duration Subgroup of PSQI.tif]

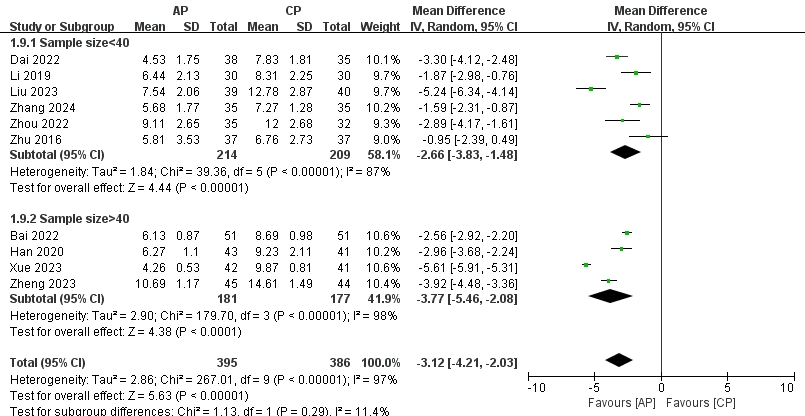

Supplement: Supplementary file 1 [file Data_Sheet_1.zip › Subgroup analysis/Subgroup analysis of PSQI/Sample size Subgroup of PSQI.tif]
